# Association of the *TNFRSF1B*-rs1061622 variant with nonresponse to infliximab in ulcerative colitis

**DOI:** 10.1038/s41598-025-02463-4

**Published:** 2025-05-25

**Authors:** Laurence Tessier, Ann-Lorie Gagnon, Sophie St-Amour, Mathilde Côté, Catherine Allard, Mathieu Durand, Danny Bergeron, Alexandre Lavoie, Alban Michaud-Herbst, Karine Tremblay

**Affiliations:** 1 Pharmacology–Physiology Department, Université de Sherbrooke, Saguenay, QC Canada; 2 Centre de recherche et d’innovation du Centre intégré universitaire de santé et de services sociaux du Saguenay–Lac-Saint-Jean (CIUSSS-SLSJ), 225, St-Vallier Street Pavillon des Augustines, Saguenay, QC G7H 7P2 Canada; 3 Centre de recherche du Centre Hospitalier Universitaire de Sherbrooke (CR-CHUS), Sherbrooke, QC Canada; 4 Unité de recherche clinique et épidémiologique (URCE), Centre de recherche du Centre, Hospitalier Universitaire de Sherbrooke (CRCHUS), Sherbrooke, Québec, CA Canada; 5 RNomics platform, Université de Sherbrooke, Sherbrooke, QC Canada; 6 Pharmacy Department, Centre Intégré Universitaire de Santé et de Services Sociaux du Saguenay–Lac-Saint-Jean (Chicoutimi University Hospital), Saguenay, QC Canada; 7 Gastroenterology Department, Centre Intégré Universitaire de Santé et de Services Sociaux du Saguenay–Lac-Saint-Jean (Chicoutimi University Hospital), Saguenay, QC Canada

**Keywords:** Ulcerative colitis, Pharmacogenomics, Genetic association study

## Abstract

For severe forms of ulcerative colitis (UC), a chronic inflammatory bowel disease (IBD), biological therapies, including tumor necrosis factor inhibitors (anti-TNF), are often used. However, these drugs have a high variability in treatment response. Multiple factors, such as genetic variants, can affect this variability. The goal of the study was to verify if selected candidate variants could affect response to anti-TNF in UC treatment. This association study included 76 participants suffering from UC and past or current users of anti-TNF. Clinical data for phenotyping was collected through a single visit with the participant and a medical chart review. Blood or saliva samples were collected to extract DNA and to genotype eight selected candidate variants in genes *TNF*, *TNFAIP3*, *TNFRSF1 A* and *TNFRSF1B*. For anti-TNF users, 30% of individuals were non-responders, 70% suffered from AE and none of the studied variants was associated with the response’s phenotype. However, for infliximab users only (*n* = 44), the *TNFRSF1B*-rs1061622 variant was associated with nonresponse to infliximab for the first time in a cohort of UC patients (p-value = 0.028). Next steps are to replicate this association in independent cohorts and to perform functional studies to gain more evidence on the variant.

**Supplementary Information:**

The online version contains supplementary material available at 10.1038/s41598-025-02463-4.

## Introduction

In 2023 in Canada, approximately 160,000 people were suffering from ulcerative colitis (UC)^1^, an inflammatory bowel disease (IBD) like Crohn’s disease (CD)^2^, which represents an important socio-economic burden^3^ and impact on the quality of life of people living with this disease^4^. Both UC and CD originate from a shared pathophysiology not yet entirely elucidated. Indeed, irregularities in the immune system and in the gut’s microbiome induce an unwanted elevated immune response in the digestive system which causes inflammation^5^. UC is characterized by continuous inflammation restricted to the colon that usually begins at the rectum^2^, and its principal symptoms are bleeding diarrhea, abdominal pain, with occasional nausea, vomiting and weight lost^4^. UC’s etiology is complex and multifactorial^4,6,7^, and its onset usually occurs in young adults but may occur at any age^8^.

There are currently no efficient pharmacological therapies to cure UC. The treatment goal is to reach and maintain remission by reducing inflammation of the colon^6^. Severity of UC may be assessed based on symptoms, disease extent and mucosal inflammation^9^. In mild to moderate UC, 5-aminosalicylates (5-ASA) are used to achieve treatment goal^9^. For moderate to severe UC, corticosteroids and immunosuppressants may be used to maintain remission^10^. When these conventional therapies fail, biotherapies (biologics) can be introduced in patients’ management plan^9^. Some of the most widely used biologics in UC are molecules targeting the tumor necrosis factor alpha (TNFα) pathway including adalimumab, infliximab and golimumab. One concern with the use of these anti-TNF agents is the high variability of their therapeutic response. According to estimates from previous studies, 18–50% of anti-TNF treated UC patients did not show any improvement and 50–90% did not reach remission^11,12^. Moreover, 40–90% suffered from adverse events (AE) among which, 5–20% will be severe AE (SAE)^12,13^. This high variability in response to anti-TNF accentuates the need for precision medicine to limit the unnecessary exposure to ineffective molecules and occurrence of possible AE/SAE.

Multiple factors were identified to explain anti-TNF response variability in treatment of UC, such as age, sex, weight, smoking habits, disease location, duration, and severity^14^, but increasing attention is paid on pharmacogenetic (PGx) variants associated with anti-TNF response phenotypes in IBD^15^. Among them, the most promising are those involved in anti-TNF pharmacodynamic mechanisms (summarized in Supplementary Table S1), but they were associated only in the context of CD treatment. Thus, this study aimed to assess anti-TNF response’s phenotypes of UC patients and to verify the association with the candidate PGx variants previously identified in CD.

## Results

### Subjects characteristics

**Fig. 1:**
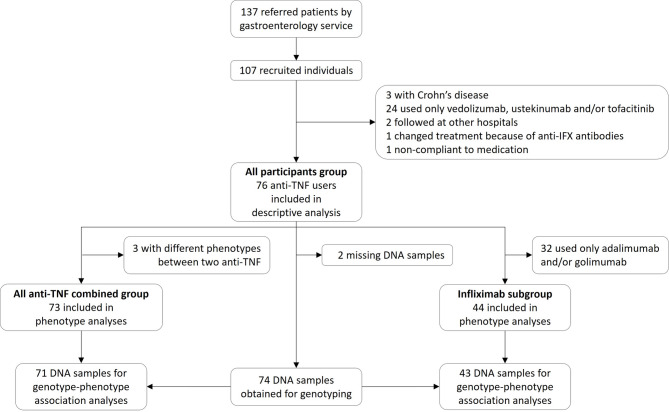
Trajectory and criteria of the participants’ selection in final analyses. 137 patients were referred by the gastroenterology department of Hôpital de Chicoutimi and 107 patients were recruited. Following medical chart review, 76 patients were successfully phenotyped (all participants group) because it was found that three of the recruited patients had Crohn’s disease instead of ulcerative colitis, 24 only used vedolizumb, ustekinumab and/or tofacitinib, two were followed at a different hospital, one changed treatment solely because of anti-infliximab antibodies and one wasn’t compliant to their medication. Two people out of the 76 did not give DNA samples du to lost to follow-up of participants who had to send saliva sample by mail. For all anti-TNF combined group analyses of phenotype association, 73 patients were included because three had different phenotypes for two different anti-TNF, and 71 of these patients gave biological sample for DNA genotyping. For the infliximab subgroup phenotype analyses, 44 patients were included because the others had only taken other anti-TNFs and 43 of these patients gave biological sample for DNA genotyping.

Among 137 UC patients treated with biotherapies followed by gastroenterologists at the “Centre intégré universitaire de santé et de services sociaux du Saguenay−Lac-St-Jean” (CIUSSS-SLSJ), 107 accepted to participate in the study. Following medical records review, 76 participants met all inclusion criteria (*all-participants group*). However, three of these participants were non-responders (NR) for a first anti-TNF and responders for a second anti-TNF, they were therefore excluded for phenotype analysis in the *all anti-TNF combined group* (*n* = 73) but kept in the *infliximab subgroup* (*n* = 44). Figure 1 summarizes participants’ selection.

Table 1 presents descriptive characteristics and phenotype frequencies of the studied groups (“all participants group”, *n* = 76; “infliximab subgroup”, *n* = 44; “participants not on infliximab subgroup”, *n* = 32). Reported sexes and genders were 100% matched for each participant (only sexes presented), and all participants were of European descent. In the all-participants group, 39 (51.3%) were women and the mean age at diagnosis was 36.7 (± 15.4) years. Additionally, 47 (61.8%) of them were former or current tobacco users and an important proportion had inflammatory comorbidities in musculoskeletal and respiratory systems (63.2% and 48.7%, respectively). Regarding the phenotypes distribution of the all anti-TNF combined group (*n* = 73), 22 (30.1%) participants were NR including 7 (31.8%) primary non-responders (PNR), 5 (22.7%) secondary non-responders (SNR) and 10 (45.5%) intermediate responders (IR). Most of the assessed variables were comparable between the infliximab subgroup and the participants not on infliximab, except for the first taken biologic which was infliximab in the first subgroup and adalimumab in the second subgroup (p-value = < 0.0001).

**Table 1:** Characteristics of ulcerative colitis patients current or former users of an anti-TNF.

	All participants group (*n* = 76)	Infliximab subgroup (*n* = 44)	Not on infliximab (*n* = 32)	*p*-value ^a^
*Demographic parameters*				
Age, mean years (SD)				
At study inclusion	49.5 (15.9)	48.7 (15.6)	50.5 (16.6)	0.662
At ulcerative colitis diagnosis	36.7 (15.4)	34.9 (14.8)	39.2 (16.2)	0.214
Sex, *n* female (%)	39 (51.3)	21 (47.7)	18 (56.2)	0.494
Body max index, mean (kg/m^2^, SD) ^b^				
At first molecule initiation	27.2 (7.0)	26.5 (4.9)	27.9 (8.9)	0.922
At study inclusion	28.9 (6.5)	27.8 (6.3)	30.5 (6.4)	0.103
*Life habits*				
Alcohol use, *n* (%) ^c^				
Never	7 (9.2)	2 (4.5)	5 (15.6)	0.404
Former	13 (17.1)	8 (18.2)	5 (15.6)	
Moderate	50 (65.8)	31 (70.5)	19 (59.4)	
Excessive	6 (7.9)	3 (6.8)	3 (9.4)	
Tobacco use, *n* (%)				
Never	29 (38.2)	18 (40.9)	11 (34.4)	0.795
Former	39 (51.3)	21 (47.7)	18 (56.2)	
Current (not daily)	0 (0)	0 (0)	0 (0)	
Current (daily)	8 (10.5)	5 (11.4)	3 (9.4)	
Drug use, *n* (%) ^d^				
Never	52 (68.4)	28 (63.6)	24 (75.0)	0.693
Former	16 (21.1)	11 (25.0)	5 (15.6)	
Current (not daily)	7 (9.2)	4 (9.1)	3 (9.4)	
Current (daily)	1 (1.3)	1 (2.3)	0 (0)	
Cannabis use, *n* (%)				
Never	58 (79.5)	32 (74.4)	26 (86.7)	0.672
Former	10 (13.7)	7 (16.3)	3 (10.0)	
Current (not daily)	4 (5.5)	3 (7.0)	1 (3.3)	
Current (daily)	1 (1.4)	1 (2.3)	0 (0)	
Diet score, mean (SD) ^e^	6.4 (1.9)	6.7 (1.8)	6.0 (2.0)	0.170
Active physically, *n* (%) ^f^	48 (63.2)	30 (68.2)	18 (56.2)	0.340
*Medical parameters*				
Disease extent at diagnosis, *n* (%) ^b^				
E1 − Proctitis	8 (13.8)	2 (5.7)	6 (26.1)	0.103
E2 − Left-sided colitis	27 (46.6)	18 (51.4)	9 (39.1)	
E3 − Pancolitis	23 (39.7)	15 (42.9)	8 (34.8)	
Comorbidities, *n* (%)				
Gastrointestinal	55 (72.4)	30 (68.2)	25 (78.1)	0.438
Musculoskeletal	48 (63.2)	26 (59.1)	22 (68.8)	0.473
Allergies	41 (53.9)	25 (56.8)	16 (50.0)	0.643
Cardiovascular	42 (55.3)	26 (59.1)	16 (50.0)	0.488
Mental Health	37 (48.7)	23 (52.3)	14 (43.8)	0.494
Respiratory	37 (48.7)	22 (50.0)	15 (46.9)	0.820
Urogenital	39 (51.3)	22 (50.0)	17 (53.1)	0.820
Metabolic	27 (35.5)	15 (34.1)	12 (37.5)	0.811
Hepatic	25 (32.9)	12 (27.3)	13 (40.6)	0.323
Cancer	10 (13.2)	3 (6.8)	7 (21.9)	0.085
Nervous system	9 (11.8)	4 (9.1)	5 (15.6)	0.480
Other	46 (60.5)	25 (56.8)	21 (65.6)	0.483
*Treatment history*				
Length of exposure, mean (months, SD)	80.4 (55.7)	94.7 (62.4)	55.2 (38.4)	NA ^h^
Naïve to biologics, *n* (%) ^g^	71 (93.4)	35 (79.5)	32 (100.0)	NA ^h^
First biologic used, *n* (%) ^g^				
Adalimumab	30 (39.5)	4 (9.1)	26 (81.2) *^1,2^	**< 0.0001**
Infliximab	35 (46.1)	35 (79.5)	0 (0) *^1,3^	
Vedolizumab	5 (6.6)	5 (11.4)	0 (0) *^2,4^	
Golimumab	6 (7.9)	0 (0)	6 (18.8) *^3,4^	
Number of biologics, *n* (%) ^g^				
Before drug initiation				
0	71 (93.4)	35 (79.5)	32 (100.0)	NA ^h^
1	4 (5.3)	6 (13.6)	0 (0)	
2	1 (1.3)	3 (6.8)	0 (0)	
Total during ulcerative colitis treatment				
1	46 (60.5)	27 (61.4)	19 (59.4)	0.077
2	16 (21.1)	6 (13.6)	10 (31.2)	
3 or more	14 (18.4)	11 (25.0)	3 (9.4)	
Concomitant medication, *n* (%)				
5-aminosalicylic acid				
At initiation only	15 (19.7)	7 (15.9)	8 (25.0)	NA ^h^
During follow-up only	9 (11.8)	8 (18.2)	3 (9.4)	
At initiation and during follow-up	16 (21.1)	12 (27.3)	5 (15.6)	
Never	36 (47.4)	17 (38.6)	16 (50.0)	
Corticosteroids				
At initiation only	17 (22.4)	7 (15.9)	8 (25.0)	NA ^h^
During follow-up only	17 (22.4)	8 (18.2)	7 (21.9)	
At initiation and during follow-up	14 (18.4)	15 (34.1)	5 (15.6)	
Never	28 (36.8)	14 (31.8)	12 (37.5)	
Immunosuppressant				
At initiation only	15 (19.7)	13 (29.5)	3 (9.4)	NA ^h^
During follow-up only	10 (13.2)	8 (18.2)	2 (6.2)	
At initiation and during follow-up	4 (5.3)	2 (4.5)	2 (6.2)	
Never	47 (61.8)	21 (47.7)	25 (78.1)	
Colectomy, *n* (%)	7 (9.2)	4 (9.1)	3 (9.4)	1.000
*Laboratory results*				
CRP > 5 mg/L at diagnosis, *n* (%) ^b^	14 (70.0)	7 (70.0)	7 (70.0)	1.000
CRP > 5 mg/L at drug initiation, *n* (%) ^b, i^	41 (71.9)	27 (81.8)	14 (58.3)	NA ^h^
Positive to anti-drug antibodies, *n* (%) ^b, j^	18 (50.0)	17 (51.5)	1 (50.0)	NA ^h^
*Phenotypes*				
Toxicity, *n* (%)				
Adverse events ^k^	53 (69.7)	35 (79.5)	21 (65.6)	NA ^h^
Efficacy, *n* (%)				
Responders	51 (69.9)	34 (77.3)	19 (59.4)	NA ^h^
All non-responders	22 (30.1)	10 (22.7)	13 (40.6)	
Primary non-responders	7 (31.8)	3 (30.0)	4 (30.8)	
Secondary non-responders	5 (22.7)	4 (40.0)	2 (15.4)	
Intermediate responders	10 (45.5)	3 (30.0)	7 (53.8)	

Abbreviations used: CRP = C-reactive protein, SD = Standard deviation, TNF = Tumor necrosis factor alpha.

^a^ Bold numbers indicate significance of Fisher test and asterisks (*) indicate significance of Fisher *post-hoc*. Pair of categories are identified by same number beside the asterisk.

^b^ Proportion/mean/SD calculated on available data.

^c^ Moderate use is considered as less than 15 drinks per week and excessive use is considered as higher than 15 drinks per week, as per Quebec Government’s recommendations.

^d^ Drugs included: cannabis, cocaine, methamphetamine, amphetamine, phencyclidine, codeine, lysergic acid diethylamide, psilocybin and psilocin.

^e^ Self-assessed score based on attention paid to alimentation at study inclusion (1 = no attention, 10 = greatest attention).

^f^ Active is defined as > 150 min of moderate activity or > 75 min of intense activity per week (World Health Organisation criteria).

^g^ Includes biologics and tofacitinib, a small molecule used as second line medication in ulcerative colitis.

^h^ Different definitions for variables between the all participants group and the infliximab subgroup makes it impossible to compare these variables (since individual may have taken more than one anti-TNF).

^I^ CRP within 4 months before molecule initiation.

^j^ Anti-drug antibodies level of 10 AU/mL or more.

^k^ At least one adverse event self-reported or collected in the medical chart.

### Characteristics associated with nonresponse to anti-TNF in UC

#### All anti-TNF combined group

All characteristics were tested for their potential influence on anti-TNF response (NR vs. responders) and five were significantly associated in the all anti-TNF combined group (Table 2). First used biologic was associated with nonresponse to anti-TNF (p-value = 0.028). Specifically, 14 (63.6%) individuals who started with adalimumab were NR compared to 5 (22.7%) for people starting with infliximab as first biologic (*post hoc* p-value = 0.038). The length of exposure, the use of two or more biologics throughout UC treatment, concomitant use of corticosteroids and undergoing a colectomy were also associated with nonresponse to anti-TNF (p-values = < 0.0001, < 0.0001, 0.001 and 0.023, respectively). When categories of nonresponse phenotypes were compared (PNR vs. SNR vs. IR vs. responders), the association remained significant for the length of exposure, the total number of biologics during UC treatment, corticosteroids use and colectomy (Supplementary Table S3). For the first biologic taken, the association was close to significance level (p-value = 0.055).

**Table 2:** Characteristics associated with nonresponse to anti-TNF and infliximab users in ulcerative colitis patients.

	All anti-TNF combined group				Infliximab subgroup		
	*R* (*n* = 51)	NR ^a^ (*n* = 22)		*p*-value ^b^	*R* (*n* = 34)	NR ^a^ (*n* = 10)	*p*-value ^b^
*Demographic parameters*							
Age, mean years (SD)							
At study inclusion	50.3 (15.8)		46.6 (16.4)	0.424	50.4 (15.3)	42.9 (16.2)	0.207
At ulcerative colitis diagnosis	37.5 (15.2)		33.5 (15.3)	0.277	36.4 (14.8)	29.8 (14.5)	0.206
Sex, *n* female (%)	25 (49.0)		14 (63.6)	0.311	14 (41.2)	7 (70.0)	0.155
Body mass index, mean (SD) ^c^							
At first molecule initiation	27.3 (7.8)		26.4 (5.5)	0.836	27.1 (4.8)	25.3 (5.1)	0.186
At study inclusion	28.6 (7.0)		29.5 (5.7)	0.618	28.3 (7.0)	26.1 (2.9)	0.204
*Life habits*							
Alcohol use, *n* (%) ^d^							
Never	6 (11.8)		1 (4.5)	0.865	2 (5.9)	0 (0)	0.460
Former	8 (15.7)		3 (13.6)		5 (14.7)	3 (30.0)	
Moderate	33 (64.7)		16 (72.7)		25 (73.5)	6 (60.0)	
Excessive	4 (7.8)		2 (9.1)		2 (5.9)	1 (10.0)	
Tobacco use, *n* (%)							
Never	18 (35.3)		10 (45.5)	0.762	14 (41.2)	4 (40.0)	0.682
Former	27 (52.9)		10 (45.5)		17 (50.0)	4 (40.0)	
Current (not daily)	0 (0)		0 (0)		0 (0)	0 (0)	
Current (daily)	6 (11.8)		2 (9.1)		3 (8.8)	2 (20.0)	
Drug use, *n* (%) ^e^							
Never	35 (68.6)		15 (68.2)	0.302	21 (61.8)	7 (70.0)	0.367
Former	12 (23.5)		3 (13.6)		10 (29.4)	1 (10.0)	
Current (not daily)	3 (5,9)		4 (18.2)		2 (5.9)	2 (20.0)	
Current (daily)	1 (2.0)		0 (0)		1 (2.9)	0 (0)	
Cannabis use, *n* (%)							
Never	40 (81.6)		16 (76.2)	0.213	25 (75.8)	7 (70.0)	0.363
Former	7 (14.3)		2 (9.5)		6 (18.2)	1 (10.0)	
Current (not daily)	1 (2,0)		3 (14.3)		1 (3.0)	2 (20.0)	
Current (daily)	1 (2.0)		0 (0)		1 (3.0)	0 (0)	
Diet score, mean (SD) ^f^	6.3 (1.8)		6.4 (2.2)	0.931	6.4 (1.8)	7.5 (1.7)	0.196
Active physically, *n* (%) ^g^	33 (64.7)		13 (59.1)	0.792	24 (70.6)	6 (60.0)	0.701
*Medical parameters*							
Disease extent at diagnosis, *n* (%) ^c^							
E1 − Proctitis	3 (7.9)		5 (29.4)	0.129	1 (3.7)	1 (12.5)	0.657
E2 − Left-sided colitis	18 (47.4)		7 (41.2)		14 (51.9)	4 (50.0)	
E3 − Pancolitis	17 (44.7)		5 (29.4)		12 (44.4)	3 (37.5)	
Comorbidities, *n* (%)							
Gastrointestinal	36 (70.6)		18 (81.8)	0.393	23 (67.6)	7 (70.0)	1.000
Musculoskeletal	32 (62.7)		14 (63.6)	1.000	19 (55.9)	7 (70.0)	0.489
Allergies	28 (54.9)		12 (54.5)	1.000	17 (50.0)	8 (80.0)	0.148
Cardiovascular	27 (52.9)		12 (54.5)	1.000	19 (55.9)	7 (70.0)	0.489
Mental Health	26 (51.0)		11 (50.0)	1.000	17 (50.0)	6 (60.0)	0.724
Respiratory	25 (49.0)		10 (45.5)	0.804	18 (52.9)	4 (40.0)	0.721
Urogenital	26 (51.0)		11 (50.0)	1.000	15 (44.1)	7 (70.0)	0.281
Metabolic	18 (35.3)		7 (31.8)	1.000	10 (29.4)	5 (50.0)	0.271
Hepatic	15 (29.4)		9 (40.9)	0.418	9 (26.5)	3 (30.0)	1.000
Cancer	8 (15.7)		2 (9.1)	0.713	2 (5.9)	1 (10.0)	0.548
Nervous system	5 (9.8)		3 (13.6)	0.691	3 (8.8)	1 (10.0)	1.000
Other	31 (60.8)		13 (59.1)	1.000	19 (55.9)	6 (60.0)	1.000
*Treatment history*							
Length of exposure, mean (months, SD)	100.5 (53.1)		32.6 (32.6)	**< 0.0001**	113.4 (57.3)	31.0 (27.4)	**0.0001**
Naïve to biologics, *n* (%) ^h^	47 (92.2)		21 (95.5)	1.000	29 (85.3)	6 (60.0)	0.175
First biologic taken, *n* (%) ^h^							
Adalimumab	15 (29.4)		14 (63.6) *^1^	**0.028**	1 (2.9)	3 (30.0)	**0.046** ^**i**^
Infliximab	28 (54.9)		5 (22.7) *^1^		29 (85.3)	6 (60.0)	
Vedolizumab	4 (7.8)		1 (4.5)		4 (11.8)	1 (10.0)	
Golimumab	4 (7.8)		2 (9.1)		0 (0)	0 (0)	
Number of biologics, *n* (%) ^h^							
Before drug initiation							
0	47 (92.2)		21 (95.5)	1.000	29 (85.3)	6 (60.0)	0.132
1	3 (5.9)		1 (4.5)		3 (8.8)	3 (30.0)	
2	1 (2.0)		0 (0)		2 (5.9)	1 (10.0)	
Total during UC treatment							
1	44 (86.3)		2 (9.1) *^1,2^	**< 0.0001**	27 (79.4)	0 (0) *^1,2^	**< 0.0001**
2	5 (9.8)		10 (45.5) *^1^		3 (8.8)	3 (30.0) *^1^	
3 or more	2 (3.9)		10 (45.5) *^2^		4 (11.8)	7 (70.0) *^2^	
Concomitant medication, *n* (%)							
5-aminosalicylic acid							
At initiation only	11 (21.6)		4 (18.2)	0.627	7 (20.6)	0 (0)	0.084
During follow-up only	7 (13.7)		2 (9.1)		5 (14.7)	3 (30.0)	
At initiation and during follow-up	7 (13.7)		6 (27.3)		7 (20.6)	5 (50.0)	
No use	26 (51.0)		10 (45.5)		15 (44.1)	2 (20.0)	
Corticosteroids							
At initiation only	14 (27.5)		3 (13.6)	**0.001**	7 (20.6)	0 (0)	0.054
During follow-up only	6 (11.8)		9 (40.9) *^1^		5 (14.7)	3 (30.0)	
At initiation and during follow-up	6 (11.8)		7 (31.8) *^2^		9 (26.5)	6 (60.0)	
No use	25 (49.0)		3 (13.6) *^1,2^		13 (38.2)	1 (10.0)	
Immunosuppressant							
At initiation only	13 (25.5)		2 (9.1)	0.303	11 (32.4)	2 (20.0)	0.836
During follow-up only	7 (13.7)		2 (9.1)		6 (17.6)	2 (20.0)	
At initiation and during follow-up	2 (3.9)		1 (4.5)		2 (5.9)	0 (0)	
No use	29 (56.9)		17 (77.3)		15 (44.1)	6 (60.0)	
Colectomy, *n* (%)	2 (3.9)		5 (22.7)	**0.023**	1 (2.9)	3 (30.0)	**0.032**
*Laboratory results*							
CRP > 5 mg/L at diagnosis, *n* (%) ^c^	8 (72.7)		5 (62.5)	1.000	4 (66.7)	3 (75.0)	1.000
CRP > 5 mg/L at drug initiation, *n* (%) ^c, j^	26 (72.2)		13 (72.2)	1.000	19 (82.6)	8 (80.0)	1.000
Positive to anti-drug antibodies, *n* (%) ^c, k^	14 (53.8)		2 (28.6)	0.398	15 (53.6)	1 (20.0)	0.335
*Toxicity*							
Adverse events, *n* (%) ^l^	39 (76.5)		13 (59.1)	0.163	28 (82.4)	7 (70.0)	0.402

Abbreviations used: CRP = C-reactive protein, IR = intermediate responders; NR = non-responders; R = responders; PNR = primary non-responders; SNR = secondary non-responders, SD = Standard deviation, TNF = Tumor necrosis factor alpha.

^a^ Includes primary non-responders, secondary non-responders and intermediate responders.

^b^ P-value of Fisher exact test comparing responders and all non-responders combined. Bold numbers indicate significance of Fisher test and asterisks (*) indicate significance of Fisher *post-hoc*. Pair of categories are identified by same number beside the asterisk.

^c^ Proportion/mean/SD calculated on available data.

^d^ Moderate use is considered as less than 15 drinks per week and excessive use is considered as higher than 15 drinks per week, as per Quebec Government’s recommendations.

^e^ Drugs included: cannabis, cocaine, methamphetamine, amphetamine, phencyclidine, codeine, lysergic acid diethylamide, psilocybin and psilocin.

^f^ Self-assessed score based on attention paid to alimentation at study inclusion (1 = no attention, 10 = greatest attention).

^g^ Active is defined as > 150 min of moderate activity or > 75 min of intense activity per week (World Health Organisation criteria).

^h^ Includes biologics and tofacitinib, a small molecule used as second line medication in ulcerative colitis.

^i^ No significant categories following fisher *post hoc* tests.

^j^ CRP within 4 months before molecule initiation.

^k^ Anti-drug antibodies level of 10 AU/mL or more.

^l^ At least one adverse event self-reported or collected in the medical chart.

#### Infliximab subgroup

All characteristics were also tested for their influence on infliximab response in the infliximab subgroup (NR vs. responders; Table 2). Length of exposure, first taken biologic, total number of biologics during UC treatment and undergoing a colectomy were also significantly associated with nonresponse to infliximab (p-values = 0.0001, 0.046, < 0.0001 and 0.032, respectively). Analyses were also conducted for categories of nonresponse phenotypes (PNR vs. SNR vs. IR vs. responders) despite small sample sizes (Supplementary Table S3). Length of exposure, total number of biologics used throughout UC treatment, the first biologic taken and undergoing a colectomy were also associated with categories of nonresponse phenotypes to infliximab (p-values = 0.0004, 0.020, < 0.0001 and 0.032, respectively). Additionally, being naïve to biologics and the number of biologics taken before infliximab initiation were associated with categories of nonresponse phenotypes to infliximab in UC (p-values = 0.032 and 0.048, respectively).

### Adverse events distribution between phenotypes of response

#### All anti-TNF combined group

Adverse events were not associated with nonresponse to anti-TNF in UC treatment (Table 3). However, infections were more frequent in responders to anti-TNF, but the association did not remain significant after Holm-Bonferroni correction for multiple AEs testing. Similar results were observed for categories of nonresponse phenotypes to anti-TNF (Supplementary Table S4).

**Table 3:** Distribution of adverse events between response’s phenotypes to anti-TNF and infliximab users in ulcerative colitis treatment.

Reaction type, *n* (%) ^a^	All anti-TNF combined group				Infliximab subgroup			
	*R* (*n* = 51)	NR ^b^ (*n* = 22)	*p*-value ^c^		*R* (*n* = 34)	NR ^b^ (*n* = 10)	*p*-value ^c^	
			Un-Adj	Adj			Un-Adj	Adj
Neurological ^d^	18 (35.3)	4 (18.2)	0.174	1.000	14 (41.2)	2 (20.0)	0.283	1.000
Skin ^e^	14 (27.5)	7 (31.8)	0.781	1.000	9 (26.5)	2 (20.0)	1.000	1.000
Infections ^f^	10 (19.6)	0 (0)	**0.027**	0.189	7 (20.6)	0 (0)	0.177	1.000
Musculoskeletal ^g^	8 (15.7)	3 (13.6)	1.000	1.000	6 (17.6)	1 (10.0)	1.000	1.000
Site injection ^h^	6 (11.8)	5 (22.7)	0.289	1.000	0 (0)	0 (0)	1.000	1.000
Gastrointestinal ^i^	5 (9.8)	1 (4.5)	0.661	1.000	4 (11.8)	0 (0)	0.559	1.000
Cardiovascular ^j^	3 (5.9)	1 (4.5)	1.000	1.000	2 (5.9)	1 (10.0)	0.548	1.000
Respiratory ^k^	3 (5.9)	1 (4.5)	1.000	1.000	3 (8.8)	1 (10.0)	1.000	1.000
Other ^l^	27 (52.9)	11 (50.0)	1.000	1.000	22 (64.7)	6 (60.0)	1.000	1.000

Abbreviations used: Adj = adjusted; NR = non-responders; R = responders; Un-adj = un-adjusted.

^a^ Proportion calculated on total available data.

^b^ Includes primary non-responders, secondary non-responders and intermediate responders.

^c^ Bold numbers indicate significance of Fisher test. Adjusted p-value by Holm-Bonferroni correction.

^d^ Includes vertigo, dizziness, and headaches.

^e^ Includes urticarial, psoriasis, eczema, erythema, pruritus, and dryness.

^f^ Includes cellulitis and recurrent infections.

^g^ Includes arthralgia and myalgia.

^h^ Includes redness, swelling, itching, heat, and pain at site injection.

^i^ Includes nausea, vomiting, and dyspepsia.

^j^ Includes peripheral edema, increased blood pressure, and palpitations.

^k^ Includes exacerbated asthma and dyspnea.

^l^ Includes asthenia, alopecia, numbness, nosebleed, shivers, and weight loss.

#### Infliximab subgroup

Adverse event types were not associated with nonresponse nor to categories of nonresponse phenotypes to infliximab in UC treatment (Table 3 and Supplementary Table S4). Again, infections were more frequent for responders to infliximab, but the difference was not significant.

### Genotype analysis and distribution in the cohort

Variants were genotyped by a qPCR essay at the RNomic platform at Université de Sherbrooke (Sherbrooke, Quebec, Canada). Call rates for genotyping were 83.8% for rs1800629, 97.3% for rs3397 and 100% for the other tested variants. Results from Hardy Weinberg Equilibrium (HWE) analysis and genotype distribution in the cohort are presented in Table 4. All genotypes were at HWE following Holm-Bonferroni correction. Due to its low success rate of genotyping, variant rs1800629 from *TNF* gene was discarded from statistical analysis.

**Table 4:** Genotypes distribution of candidate variants for anti-TNF users in ulcerative colitis.

Gene	Variant	Alleles (REF > ALT)	Call rate (%)	ALT frequency	Observed genotypes (*n* = 74 ^a^)			HWE *p*-value ^b^		
					HMZ_REF_	HTZ	HMZ_ALT_	Un-Adj	Adj	
*TNF*	rs1800629	G > A	83.8	0.145	48	10	4	**0.016**		0.127
*TNFAIP3*	rs6927172	C > G	100.0	0.176	48	26	0	0.107		0.643
*TNFRSF1 A*	rs4149570	A > C	100.0	0.608	10	38	26	0.628		1.000
	rs767455	T > C	100.0	0.405	26	36	12	1.000		1.000
*TNFRSF1B*	rs1061622	T > G	100.0	0.250	40	31	3	0.533		1.000
	rs1061624 ^c^	A > G	100.0	0.581	17	28	29	0.058		0.407
	rs3397 ^d^	C > T	97.3	0.819	4	18	50	0.223		1.000
	rs97688 ^e^	T > C	100.0	0.662	10	30	34	0.439		1.000

Abbreviations used: Adj = adjusted; ALT = alternative allele; HMZ = homozygous; HTZ = heterozygous; HWE = Hardy-Weinberg equilibrium; REF = reference allele; *TNF* = Tumor necrosis factor alpha; *TNFAIP3* = Tumor necrosis factor alpha induced protein 3; *TNFRSF1A* = Tumor necrosis factor receptor super-family, member 1A; *TNFRSF1B* = Tumor necrosis factor receptor super-family, member 1B; Un-adj = un-adjusted.

^a^ Two missing samples for genotyping.

^b^ Bold numbers indicate significance of Fisher exact test for Hardy-Weinberg equilibrium with adjusted p-value for multiple testing by Holm-Bonferroni correction.

^c^ Allele T was not observed in group.

^d^ Two missing genotypes for this variant.

^e^ Allele A was not observed in group.

### PGx variants associated with nonresponse

#### All anti-TNF combined group

No variants were associated with nonresponse in the combined anti-TNF group (Table 5). Dominant (AA vs. AB/BB) and recessive (AA/AB vs. BB) models were also tested without finding any association in this group (Supplementary Table S5). Variant *TNFRSF1A-*rs767455 was associated with categories of nonresponse phenotypes to anti-TNF (codominant p-value = 0.015; recessive p-value = 0.022, Supplementary Table S6) but the association was lost following Holm-Bonferroni correction.

**Table 5:** Genotype distribution between response’s phenotypes to anti-TNF and infliximab in ulcerative colitis treatment.

Genotypes *n* (%) ^a^	All anti-TNF combined group				Infliximab subgroup			
	*R* (*n* = 49)	NR ^b^ (*n* = 22)	*p*-value		*R* (*n* = 33)	NR ^b^ (*n* = 10)	*p*-value ^c^	
			Un-Adj	Adj			Un-adj	Adj
*TNFAIP3*								
rs6927172								
CC	33 (67.3)	13 (59.1)	0.594	1.000	22 (66.7)	4 (40.0)	0.158	1.000
CG	16 (32.7)	9 (40.9)			11 (33.3)	6 (60.0)		
GG	0 (0)	0 (0)			0 (0)	0 (0)		
*TNFRSF1 A*								
rs4149570								
AA	7 (14.3)	3 (13.6)	0.461	1.000	5 (15.2)	1 (10.0)	1.000	1.000
AC	27 (55.1)	9 (40.9)			17 (51.5)	5 (50.0)		
CC	15 (30.6)	10 (45.5)			11 (33.3)	4 (40.0)		
rs767455								
TT	20 (40.8)	5 (22.7)	0.141	0.987	11 (33.3)	1 (10.0)	0.423	1.000
TC	24 (49.0)	11 (50.0)			17 (51.5)	7 (70.0)		
CC	5 (10.2)	6 (27.3)			5 (15.2)	2 (20.0)		
*TNFRSF1B*								
rs1061622								
TT	28 (57.1)	10 (45.5)	0.678	1.000	24 (72.7)	2 (20.0) *^1^	**0.004**	**0.027**
TG	19 (38.8)	11 (50.0)			9 (27.3)	7 (70.0) *^1^		
GG	2 (4.1)	1 (4.5)			0 (0)	1 (10.0)		
rs1061624								
AA	10 (20.4)	7 (31.8)	0.352	1.000	7 (21.2)	3 (30.0)	0.265	1.000
AG	17 (34.7)	9 (40.9)			10 (30.3)	5 (50.0)		
GG	22 (44.9)	6 (27.3)			16 (48.5)	2 (20.0)		
rs3397 ^d^								
CC	4 (8.5)	0 (0)	0.557	1.000	2 (6.5)	0 (0)	1.000	1.000
CT	12 (25.5)	6 (27.3)			6 (19.4)	2 (20.0)		
TT	31 (66.0)	16 (72.7)			23 (74.2)	8 (80.0)		
rs976881								
TT	7 (14.3)	3 (13.6)	0.880	1.000	5 (15.2)	0 (0)	0.137	0.956
TC	18 (36.7)	10 (45.5)			14 (42.4)	8 (80.0)		
CC	24 (49.0)	9 (40.9)			14 (42.4)	2 (20.0)		

Abbreviations used: Adj = adjusted; NR = non-responders; R = responders; *TNFAIP3* = Tumor necrosis factor alpha induced protein 3; *TNFRSF1A* = Tumor necrosis factor receptor super-family, member 1A; *TNFRSF1B* = Tumor necrosis factor receptor super-family, member 1B; Un-adj = un-adjusted.

^a^ Only the codominant model (AA vs. AB vs. BB) for combined nonresponse phenotypes is presented on this table (for the other models, see Supplementary Tables S5 and S6).

^b^ Includes primary non-responders, secondary non-responders and intermediate phenotype.

^c^ Bold numbers indicate significance of Fisher test and asterisks (*) indicate significance of Fisher *post hoc*. Pair of categories are identified by same number beside the asterisk. Adjusted p-value by Holm-Bonferroni correction.

^d^ This variant has missing genotypes. Two missing genotypes for the anti-TNF group (R, *n* = 47; NR, *n* = 22), and one missing genotype for the infliximab group (R, *n* = 31; NR, *n* = 10).

#### Infliximab subgroup

In the infliximab subgroup analysis, variant *TNFRSF1B-*rs1061622 was associated with nonresponse to infliximab using the codominant model and the association remained significant following Holm-Bonferroni correction (adjusted p-value = 0.027, Table 5). In a simple logistic model, this association means that for each additional G allele of the *TNFRSF1B-*rs1061622 variant (NC_000001.11:g.12192898 T > G), the odds of being a NR to infliximab are multiplied by 10 (OR = 10.4, IC 95%: [2.3–76.2]). This variant was also associated in the dominant model with Holm-Bonferroni correction (adjusted p-value = 0.049), but it was not associated in the recessive model (Supplementary Table S5). The same variant was also associated with nonresponse subphenotypes (PNR, SNR, IR), despite the small sample size for each phenotype and the application of Holm-Bonferroni correction, in both codominant and dominant models (adjusted p-values = 0.035 and 0.042, respectively, Supplementary Table S6). No other association was found between nonresponse to infliximab and other tested variants.

## Discussion

Our results show the first association of *TNFRSF1B*-rs1061622 variant with nonresponse to infliximab in a cohort of UC patients. However, none of the studied variants were associated with any phenotypes of response in the combined anti-TNF cohort. Combining anti-TNF is often used in numerous studies to increase sample size^16–21^. This practice may not be the ideal way to analyse nonresponse to these medications because, despite having the same pharmacological mechanism of action, infliximab, adalimumab and golimumab are three distinct drugs, and can therefore be affected by different PGx variants.

Our results support this hypothesis by the finding that adalimumab used as first biologic was associated with nonresponse when compared to infliximab (*post hoc* p-value = 0.038). Ananthakrishnan *et al.* also showed a trend towards a higher likelihood to be a NR for adalimumab compared to infliximab^22^. These observed differences might only be due to chance but could also be due to the molecular differences between the two treatments. Despite being both anti-TNF, the treatment response to these two drugs could be impacted by different biological factors because, for instance, they bind to TNFα differently^23^.

Corticosteroids concomitant use has been associated with nonresponse to infliximab^12^ and to a higher risk of colectomy in infliximab UC users^24,25^. The present study was able to replicate the association with nonresponse in the all anti-TNF combined group, but not in the infliximab subgroup. However, the use of corticosteroids is indicated in case of active diseases in UC guidelines^26^ and is often used as a treatment goal (steroid-free remission) to illustrate a persistent state of remission^9^. Therefore, it’s use may be induced by the nonresponse itself. Nevertheless, the mechanisms by which the use of corticosteroids during anti-TNF treatment could affect drug response need further investigations to better define and understand this possible drug-drug interaction.

Total number of biologics used in UC treatment and undergoing a colectomy were two variables associated with nonresponse to anti-TNF. For the NR to anti-TNF, the use of another biologic is indicated to obtain remission, and when all the therapeutic options have failed or inflammation state is extreme, the last treatment option is surgery according to UC treatment guidelines^9^. These associations with nonresponse to anti-TNF represent a high burden for the health system, for patients who cannot achieve remission at first biological agent, and for those who could never achieve remission and had a colectomy. Therefore, it becomes pressing to study this population of NR to the available treatments to guide the development of new drugs, and to establish methods to determine the right medication for patients at initiation to achieve a faster remission.

Responders had a significant longer length of exposure to therapies than non-responders to anti-TNF which is expected since responder are less likely to discontinue their treatment.

Our result showed no significant association between specific AE and response phenotypes to anti-TNF. However, infections were observed only in responders to anti-TNF, but the small number of AE identified may have prevented us from finding a significant association. To our knowledge, this is the first study to compare efficacy to toxicity of anti-TNF in the treatment of UC.

The variant *TNFRSF1B-*rs1061622 was associated with nonresponse to infliximab in UC in this study. In a German study performed with two cohorts, the alternative allele (G) was associated with nonresponse to infliximab in CD only in their first cohort^27^. In a Dutch study, the alternative allele was associated with a better response to infliximab in CD compared to PNR^28^. Similar results were observed in a Spanish study in which the alternative allele has been associated with remission of CD patients^29^. However, two other studies (one Japanese and one Flemish) did not find any association between the variant and response to infliximab after four weeks of treatment in CD^30,31^. All these studies were conducted on CD, none was reported for UC. Therefore, the present study is the first to assess and to report an association between the variant *TNFRSF1B-*rs1061622 and nonresponse to infliximab. This variant induces a nucleotide change from thymine (T, reference allele) to guanine (G, alternative allele) which translates to a change in the 196th amino acid from methionine (M) to arginine (R)^32^. The amino acid substitution is located in the fourth cysteine-rich motif of the extracellular domain (binding site of TNFα) of the TNFα receptor 2 (TNFR2)^33^. Despite the location of the genetic variation, the affinity of this receptor with its natural ligand TNFα does not seem to be affected by this mutation^34,35^. However, a study showed an increase production of interleukin 6 (IL-6) in *TNFRSF1B-*rs1061622(G) HeLa cell lines, compared to the *TNFRSF1B-*rs1061622(T) HeLa cell lines^35^. IL-6 has been shown to be increased in IBD patients and inhibited by anti-TNF treatments^36^. In inflammatory process, membrane bound TNF-alpha (mTNFα) binds to TNFR2, which induces recruitment of the TNF receptor associated factor 2 (TRAF2) to the receptor^37^. Following TRAF2 binding, nuclear factor-kappa B (NFκB) pathway is activated resulting in an increased transcription of multiple cytokines, including IL-6^37^. IL-6 functions are mainly to maintain T cell integrity and to resist apoptosis, therefore increasing inflammation by maintaining these cells alive^38^. Thus, an increase IL-6 expression due to *TNFRSF1B-*rs1061622(G) could be an avenue to explore to better understand the effect of the variant on treatment response.

Small sample size is an inherent limitation for all genetic studies. However, since UC affects less than 1% of Canadians^39^ and that only 5 to 15% of these will be using a biologic during the course of their treatment^40^, the number of patients included in the study was a convenient sample among all possible cases in our SLSJ region (282,330 inhabitants in 2023^41^, meaning that ~ 2,800 prevalent cases are expected, including ~ 230 cases on biotherapies, all followed in one central hospital deserving gastroenterology specialty). Thus, despite the small sample size of our cohort, we found significant association even when applying stringent correction for multiple testing, which highlights the strength of our deep phenotyping strategy^12^ combined with a targeted candidate-gene approach and the advantages of the use of a well-known founder population in genetic studies^42–48^. However, by using such cohort of 100% European descent, the results cannot be generalized to general admixed populations. Results must be replicated in other cohorts and associated variant studied in diverse ethnic groups in future studies. Grouping all anti-TNF agents together was another limitation of the study because these drugs, despite having the same action mechanism, may be affected by different PGx variants. Fortunately, the infliximab subgroup had enough participants for us to perform stratification analyses. Replication studies could consider analyzing anti-TNF separately to identify specific PGx variants for each molecule. Combining retrospective and observational data collection in the experimental design also strengthened the study because, for instance, during a single observational visit, participants may forget information (e.g. a specific concomitant disease or medication) which can be found in the medical records review. Conversely, the medical records review may not consign information on lifestyle habits, thus the information collected during the patient’s interview empowered the depth and quality of our dataset.

## Conclusion

This study reported the first association between *TNFRSF1B*-rs1061622 variant and nonresponse to infliximab in the treatment of UC. The functional mechanisms underlying this association remain unclear and need to be investigated more deeply. Furthermore, replication association studies in independent cohorts are mandatory to increase the level of evidence before it can become a predictive marker of nonresponse to guide and help the choice of treatment in UC.

## Methods

### Study population and inclusion criteria

This genetic association study was conducted at the CIUSSS-SLSJ, a university hospital located in the SLSJ region of the province of Quebec (Canada). To participate, patients had to be 18 years old or more, have received a UC diagnostic, be followed by the CIUSSS-SLSJ gastroenterology service, be current or former users of one of the three studied anti-TNF (adalimumab, infliximab or golimumab) during at least one year and be compliant to their prescribed regimen. All participants originate from SLSJ, a well-recognized founder population^44^.

### Recruitment

The CIUSSS-SLSJ gastroenterology service referred patients who had consented to be contacted for research purposes by delegated team members (LT, ALG, SSA, MC). For the individuals interested in participating to the study, following free and informed consent, questionnaires collecting medical history, lifestyle habits and molecule response were completed during a single visit (in person or online). Blood (approximately 10 mL) or saliva (approximately 4 mL) samples were then collected to isolate DNA depending on participants’ preference. All information was collected directly on REDCap, an electronic data capture tools hosted by Université de Sherbrooke^49,50^.

### Data collection

Following participant’s visit, the medical records were reviewed to assess the anti-TNF’s response phenotypes from UC diagnosis up to the same end date for each participant (January 1st, 2023). Data entries included start and end dates of each anti-TNF taken, symptoms evolution, concomitant medication taken throughout the treatment, endoscopic results, lab results such as C-reactive protein (CRP) and anti-drug antibodies, and medical personal history. Endoscopic results were evaluated with the Mayo score, a standardized evaluation score for endoscopies^51^. For the Mayo endoscopic subscore that was not found in the medical records, one gastroenterologist (AMH) scored each of these endoscopies. A 10% proportion of medical records was validated to assure data quality (discrepancies found were low, approximately 4%).

### Response phenotype definitions

Response phenotypes were defined based on our previously defined criteria^12^. In brief, individuals who had uninterrupted treatment for at least 12 months and whose last endoscopic evaluation revealed a total Mayo score of 0 or 1 were defined as responders. Those who could not achieve clinical nor endoscopic remission (defined as a Mayo endoscopic subscore of 0 or 1) and who did not observe any endoscopic improvement nor decrease in symptoms were defined as primary non-responders (PNR). Individuals who observed a period of remission and then a relapse of symptoms and could not reattain remission afterwards (loss of response, LOR) were considered as secondary non-responders (SNR). Finally, the ones who could not be classified between these three categories were classified as intermediate responders (IR).

AE were self-reported during interviews with participants or retrieved from medical records.

### Genetic variants selection

A literature review was completed using PubMed^®^ and PharmGKB^52^ to identify candidate genes that had been associated with anti-TNF response in UC, CD or combined IBD. Variants were selected for their association with anti-TNF response in IBD, their link to the pharmacodynamics of anti-TNF and a minor allele frequency higher than 5% (Supplementary Table S1). Details on selected genes and variants can be found in Supplementary Methods. All selected variants had already been associated to phenotype of response to anti-TNF in CD, but not in UC.

### Genotype

Blood samples were drawn in EDTA tubes and buffy coat was isolated within 24 h. DNA was extracted from buffy coat using the Puregene Blood Kit (QIAGEN, Germany). For saliva samples, DNA was extracted directly from samples received by postal mail from participants in GenoTech^®^ saliva sample collection kit OG-500 and using the prepIT-L2P for extraction (DNAgenoteck, Ottawa, Canada). DNA samples were genotyped by qPCR assays at the Université de Sherbrooke RNomics platform lab by research professionals (MD, DB) and team member (LT). For the qPCR assays, primers (IDT) were individually resuspended to 100 µM stock solutions in Tris-EDTA buffer. qPCRs were performed in a 10 µL volume in 96-well plates on a CFX OPUS-96 thermocycler (Bio-Rad) with 5 µL of 2X Probe Supermix (prepared by the Plateforme de purification de protéines de l’Université de Sherbooke), 10 ng (3 µL) of DNA, 250 nM (final concentration; 0.025 µL) primer pair solutions and Probe (IDT; Affinity Plus Fam/HEX) and 1.9 µL of RNase DNase-free water (Wisent). The following cycling conditions were used: 2 min at 95 °C, 50 cycles of 15 s at 95 °C, 1 min at 60 °C. Probes and primers design used can be found in Supplementary Table S2.

### Statistical analysis

Hardy-Weinberg Equilibrium (HWE) was tested for each variant. Assessment of validity of genotyping was made based on HWE results and on genotyping call rate. Responders were compared to NR (all together) for basic characteristics, for AEs, as well as for variant genotypes. Categories of response phenotypes (4 categories: responders, PNR, SNR and IR) were also compared for the same variables. Primary analyses were done on the all anti-TNF combined group and were then repeated on the infliximab subgroup. For comparisons between groups, Wilcoxon tests (when there were two groups compared) and Kruskal-Wallis tests (when there were more than 2 groups compared) were used for continuous variable, and Fisher exact tests were used for dichotomic and categorical variables. When significant, *post hoc* tests were done on each pair of categories and each pair of phenotypes when 4 categories of response phenotypes were compared (R vs. PNR vs. SNR vs. IR). Holm-Bonferroni^53^ correction was applied for *post hoc* multiple testing. When a variant was found significant, a simple logistic regression with an additive model for the variant (unadjusted) was used to quantify the effect on the odds of being in the NR group. For variant analyses, each variant was compared using the codominant model (AA vs. AB vs. BB; where A is the reference allele and B the alternative allele based on National Center of Biotechnology Information (NCBI) classification), the dominant model (AA vs. AB/BB) and the recessive model (AA/AB vs. BB). P-value significative threshold was set to 0.05 and Holm-Bonferroni^53^ correction was used to account for multiple variants and AEs testing. All statistical analyses were done using R version 4.3.0^54^.

### Ethical considerations

The project was approved and overviewed by the Institutional Ethics Review Board of the “Centre intégré universitaire de santé et de services sociaux du Saguenay−Lac-St-Jean” (#2020-038). All research was conducted according to relevant guidelines and regulations.

## Electronic supplementary material

Below is the link to the electronic supplementary material.

Supplementary Material 1

## Data Availability

The minimal dataset that would be necessary to interpret or replicate findings of the current study may be available from the corresponding author upond request and according to local access policies.
