# Supplementary material for: Association of the TNFRSF1B-rs1061622 variant with nonresponse to infliximab in ulcerative colitis
Source: Sci Rep. 2025 May 25;15:18240. doi: 10.1038/s41598-025-02463-4 (PMC12104363; doi:10.1038/s41598-025-02463-4)
Supplement: Supplementary file 1 — Supplementary Material 1 [file 41598_2025_2463_MOESM1_ESM.docx]

**Association of the *TNFRSF1B* rs1061622 variant with nonresponse to infliximab in ulcerative colitis**

**Authors**

Laurence Tessier, B.Sc., Ann-Lorie Gagnon, M.Sc., Sophie St-Amour, B.Sc., Mathilde Côté, Catherine Allard, M.Sc., Mathieu Durand, M.Sc., Danny Bergeron, Ph.D., Alexandre Lavoie, M.Sc., Alban Michaud-Herbst, MD, and Karine Tremblay, Ph.D.

SUPPLEMENTARY MATERIAL

**Supplementary Method**

Candidate Gene Selection

Anti-TNFs target TNFα to inhibit its pro-inflammatory pathways involved in activating cytokines and chemokines^1^. In the *tumor necrosis factor* (*TNF*) gene, coding for the TNFα protein expression, genetic variation rs1800629 alternative allele (A) has been associated with nonresponse to anti-TNF in CD^2–5^ and has been shown to increase TNFα expression^6^. For these non-responders (NRs), the anti-TNF agent may not bloc enough TNFα due to its higher level, which could diminish the drug’s effect^2^.

Additionally, the genes *tumor necrosis factor receptor super family members 1A* and *1B* (*TNFRSF1A* and *TNFRSF1B*) both encode for TNFα receptors TNFR1 and TNFR2, respectively^7^. These receptors induce pro-inflammatory signals and cell death following activation by TNFα^1^. Two genetic variations in gene *TNFRSF1A*, rs4149570 and rs767455, were associated with phenotype of response to anti-TNF in CD^8–10^. For rs4149570, reference allele (A) was associated with better response to anti-TNF in CD and in a combined IBD group (including CD and UC patients)^8^ and with an increase expression of the receptor TNFR1^11^. This indicates an increase TNFα pathway which could explain why the inhibition of this pathway by anti-TNF has a greater effect on these individuals^8^. For rs767455, presence of alternative allele (C) was associated with nonresponse to infliximab in CD^9,10^ and with lower expression of TNFR1^12^. This decreased TNFα pathway may explain the reduced response to anti-TNF. For *TNFRSF1B* gene, four genetic variations, rs1061622, rs1061624, rs3397 and rs976881, were associated with response to anti-TNF in CD in previous studies^13–17^. However, most of these genetic variations were associated with both better and worst response. Result contradictions may be explained by the different methods used to evaluated response phenotypes. Nevertheless, these variants could explain part of response to anti-TNF. For example, rs3397, a 3’UTR variant, has been associated with lower TNFR2 mRNA expression for alternative allele (T), which could influence response by decreasing TNFR2 pathway^12^.

Furthermore, gene TNFAIP3 encodes a protein (A20) whose expression is TNFα-induced^18,19^, and is, therefore, another gene of interest in anti-TNF response. Alternative allele (G) of the genetic variation rs6927172 in this gene was associated with nonresponse to anti-TNF in a combined IBD cohort^8^ and was also associated with an increase expression of A20^20^. One of A20 function is to inhibit the NFᴋB pathway induced by TNFα binding to its receptors^21^. Therefore, similarly to *TNFRSF1A*- rs767455, an already decreased TNFα pathway may reduce the effect of the anti-TNF agent.

**Supplementary Tables**

**Table S1. Gene related to anti-TNF’s pharmacodynamic and previously associated with response phenotypes to anti-TNF in Crohn’s disease**

| **Gene**  **Symbol**  **[GeneID]**  **Locus** | **Variants -**  **SNV ^a^** | **Alleles**  **(REF>ALT) ^b^** | **Allele frequency [allele] ^c^** | **SNV type [amino acids]** | **Medication** | **Disease** | **Phenotype of response [allele]** | **Ethnicity (Number)** | **ref** |
| --- | --- | --- | --- | --- | --- | --- | --- | --- | --- |
| *TNF (TNFA)*  [7124]  6p21.33 | rs1800629 | G>A | 0.15 [A] | 2KB Upstream | IFX, ADA | Combined IBD | NR [A] | CAUC (34) | ^2^ |
|  |  |  |  |  | IFX, ADA, CTZ | CD | NR [A] | 93% CAUC  7% AA  (100) | ^3^ |
|  |  |  |  |  | IFX, ADA, CTZ,  ETA,  rhTNFR-Fc | Combined auto-immune disease (PSA, AS, IBD, CD) | R [G] | 80% CAUC  20% Asian  (626) | ^4^ |
|  |  |  |  |  | IFX, ADA, CTZ,  ETA,  rhTNFR-Fc | Combined auto-immune disease (PSA, AS, CD) | R [G] | CAUC  (392) | ^22^ |
| *TNFAIP3*  [7128]  6q23.3 | rs6927172 | C>G | 0.09 [G] | 2KB Upstream | IFX, ADA | Combined IBD and UC | NR [G] | CAUC (738:  256 UC/482 CD) | ^8^ |
|  |  |  |  |  | ADA | CD | NR [C] | CAUC (102) | ^23^ |
| *TNFRSF1A*  [7132]  12p13.31 | rs4149570 | A>C | 0.62 [C] | 2KB Upstream | IFX, ADA | Combined IBD and CD | R [A] | CAUC (738:  256 UC/482 CD) | ^8^ |
|  | rs767455 | T>C | 0.42 [C] | SYN | IFX | CD | NR [C] | Asian (81) | ^9^ |
|  |  |  |  |  | IFX | CD | NR [C] | CAUC (166) | ^10^ |
| *TNFRSF1B*  [7133]  1p36.22 | rs1061622 | T>G | 0.23 [G] | Missense [Met196Arg] | IFX | CD | R [G] | CAUC (124) | ^14^ |
|  | rs1061624 | A>G,T | 0.54 [G]  0.00 [T] | 3’UTR | IFX | CD | R [A] | CAUC (297) | ^15^ |
|  |  |  |  |  | IFX | CD | R [A] | CAUC (132) | ^16^ |
|  | rs3397 | C>T | 0.58 [T] | 3’UTR | IFX | CD | R [T] | CAUC (297) | ^15^ |
|  |  |  |  |  | IFX, ADA | Combined IBD | NR [T] | CAUC (209:  62 UC/147 CD) | ^17^ |
|  | rs976881 | T>C,A | 0.69 [C]  0.00 [A] | Intron | IFX | CD | LOR [T] | CAUC (124) | ^14^ |

Abbreviations used: AA = African American; ADA = Adalimumab; AS = Ankylosing spondylitis; CAUC = Caucasian; CD = Crohn’s disease; CTZ = Certolizumab; ETA = Etanercept; IBD = Inflammatory bowel disease; IFX = Infliximab; PSA = Psoriatic arthritis; rhTNFR-Fc = TNF-α receptor II–IgG Fc fusion protein; UC = Ulcerative colitis.

^a^ Data from GRCh38/hg38 version; http://ncbi.nlm.nih.gov/snp/.

^b^ The reference allele and the alternative allele are based on the National Center of Biotechnology Information (NCBI) classification.

^c^ From ALFA project

**Table S2. TaqMan PCR Primers and probes**

| **Gene**  **(variant)** | **Type ^a^** | **Design ^b^** |
| --- | --- | --- |
| *TNF* |  |  |
| rs1800629 | Primer forward | 5’-GTTAGAAGGAAACAGACCACAGA-3’ |
|  | Primer reversed | 5’-CTGGGCCACTGACTGATTT-3’ |
|  | Probe Ref G | 5’-CGTCC+CCATGCCCCTCA-3’ |
|  | Probe Alt A ^c^ | 5’-CGTC+C+T+CATGCCCCTCA-3’ |
| *TNFAIP3* |  |  |
| rs6927172 | Primer forward | 5’-GGAGCTAATCAAGTGGCAATG-3’ |
|  | Primer reversed | 5’-GGTCATTGTATTCTGAGTCTTTCTG-3’ |
|  | Probe Ref C | 5’-AAAGTTGAC+CT+G+G+AT+TTCCC-3’ |
|  | Probe Alt G ^c^ | 5’-AAAGTTGAC+CT+G+C+AT+TTCCC-3’ |
| *TNFRSF1A* |  |  |
| rs4149570 | Primer forward | 5’-GTCTTGGACACATAAATGAACTTCTC-3’ |
|  | Primer reversed | 5’-AGGAGACAGGTTATCTCCACTC-3’ |
|  | Probe Ref A | 5’-CACATAA+CTGA+A+A+CTG+TC+TGGATC-3’ |
|  | Probe Alt C ^c^ | 5’-CACATAACTGA+A+C+CTG+TCTGGATC-3’ |
| rs767455 | Primer forward | 5’-CCACCAGCCCACTCTTC-3’ |
|  | Primer reversed | 5’-GAGAGGCCATAGCTGTCTG-3’ |
|  | Probe Ref T | 5’-TCACCAG+TG+GCAGCAGCA-3’ |
|  | Probe Alt C ^c^ | 5’-TCACCAG+CGGCAGCAGCA-3’ |
| *TNFRSF1B* |  |  |
| rs1061622 | Primer forward | 5’-CTCCAGCTGTAACGTGGTG-3’ |
|  | Primer reversed | 5’-CGTGTGTTGGGATCGTGT-3’ |
|  | Probe Ref T | 5’-ATGCAA+GC+A+T+GGATGCAGTCT-3’ |
|  | Probe Alt G ^c^ | 5’-ATGCAAGC+A+G+GGATGCAGTCT-3’ |
| rs1061624 | Primer forward | 5’-TTCCTCTAGTGCCCTCCAC-3’ |
|  | Primer reversed | 5’-GCCTTCCGAGAGGGACA-3’ |
|  | Probe Ref A | 5’-AGGC+AGC+G+A+GTT+GTGGAA-3’ |
|  | Probe Alt G ^c^ | 5’-AGGCAGC+G+G+GTTGTGGAA-3’ |
|  | Probe Alt T | 5’-AGGC+AGC+G+T+GTTGTGGAA-3’ |
| rs3397 | Primer forward | 5’-GGCAGCGAGTTGTGGAA-3’ |
|  | Primer reversed | 5’-TCACAGAGAGTCAGGGACTT-3’ |
|  | Probe Ref C ^d^ | 5’-TGCCATGG+C+GTGTCCCT-3’ |
|  | Probe Alt T ^c^ | 5’-TGCCATG+G+T+GTGTCCCT-3’ |
| rs976881 | Primer forward | 5’-GGAAGCCTTGAGCAGGAAG-3’ |
|  | Primer reversed | 5’-AAGCCCTAGGCCTGGAA-3’ |
|  | Probe Ref T | 5’-CCTTT+GT+CG+C+T+GG+TAATGG-3’ |
|  | Probe Alt C ^d^ | 5’-CCTTTGT+CG+C+C+GGTAATGG-3’ |
|  | Probe Alt A ^c^ | 5’-CCTTT+GT+CG+C+A+GG+TAATGG-3’ |

Abbreviations used: ALT = alternative allele; REF = Reference allele.

^a^ All probes had quencher on 3 prime end and all reference probe (Probe Ref) had FAM fluorophore on 5 prime end.

^b^ + indicate locked nucleotide acid was use for next nucleotide.

^c^ HEX fluorophore on 5 prime end.

^d^ Cy5 fluorophore on 5 prime end.

**Table S3.** **Characteristics associated with stratified phenotype of nonresponse to anti-TNF and infliximab in ulcerative colitis treatment**

|  | **All anti-TNF combined group** | | | | |  |  | **Infliximab subgroup** | | |  |
| --- | --- | --- | --- | --- | --- | --- | --- | --- | --- | --- | --- |
|  |  | **Phenotype stratification** | | |  |  |  | **Phenotype stratification** | | |  |
|  | **R**  **(n = 51)** | **PNR**  **(n = 7)** | **SNR**  **(n = 5)** | **IR**  **(n = 10)** | **p-value ^b^** |  | **R**  **(n = 34)** | **PNR**  **(*n* = 3)** | **SNR**  **(*n* = 4)** | **IR**  **(*n* = 3)** | **p-value ^b^** |
| *Demographic parameters* |  |  |  |  |  |  |  |  |  |  |  |
| Age, mean years (SD) |  |  |  |  |  |  |  |  |  |  |  |
| At study inclusion | 50.3 (15.8) | 46.4 (19.3) | 42.0 (13.1) | 49.1 (16.9) | 0.772 |  | 50.4 (15.3) | 50.3 (22.0) | 43.0 (18.5) | 35.3 (3.2) | 0.400 |
| At UC diagnosis | 37.5 (15.2) | 35.1 (15.6) | 22.8 (8.4) | 37.8 (16.4) | 0.192 |  | 36.4 (14.8) | 36.5 (11.8) | 31.0 (19.3) | 21.4 (8.6) | 0.252 |
| Sex, *n* female (%) | 25 (49.0) | 6 (85.7) | 3 (60.0) | 5 (50.0) | 0.328 |  | 14 (41.2) | 3 (100.0) | 2 (50.0) | 2 (66.7) | 0.255 |
| Body max index, mean (SD) ^c^ |  |  |  |  |  |  |  |  |  |  |  |
| At first molecule initiation | 27.3 (7.8) | 26.2 (5.8) | 27.4 (7.3) | 26.0 (4.8) | 0.982 |  | 27.1 (4.8) | 22.6 (0.7) | 25.0 (5.9) | 28.2 (6.5) | 0.247 |
| At study inclusion | 28.6 (7.0) | 28.6 (5.9) | 30.3 (8.8) | 29.8 (4.1) | 0.790 |  | 28.3 (7.0) | 25.7 (0.7) | 25.1 (2.5) | 27.7 (4.8) | 0.400 |
| *Life habits* |  |  |  |  |  |  |  |  |  |  |  |
| Alcohol use, *n* (%) ^d^ |  |  |  |  |  |  |  |  |  |  |  |
| Never | 6 (11.8) | 0 (0) | 0 (0) | 1 (10.0) | 0.940 |  | 2 (5.9) | 0 (0) | 0 (0) | 0 (0) | 0.460 |
| Former | 8 (15.7) | 1 (14.3) | 1 (20.0) | 1 (10.0) |  |  | 5 (14.7) | 0 (0) | 2 (50.0) | 1 (33.3) |  |
| Moderate | 33 (64.7) | 5 (71.4) | 3 (60.0) | 8 (80.0) |  |  | 25 (73.5) | 2 (66.7) | 2 (50.0) | 2 (66.7) |  |
| Excessive | 4 (7.8) | 1 (14.3) | 1 (20.0) | 0 (0) |  |  | 2 (5.9) | 1 (33.3) | 0 (0) | 0 (0) |  |
| Tobacco use, *n* (%) |  |  |  |  |  |  |  |  |  |  |  |
| Never | 18 (35.3) | 3 (42.9) | 1 (20.0) | 6 (60.0) | 0.358 |  | 14 (41.2) | 0 (0) | 1 (25.0) | 3 (100.0) | 0.050 |
| Former | 27 (52.9) | 2 (28.6) | 4 (80.0) | 4 (40.0) |  |  | 17 (50.0) | 1 (33.3) | 3 (75.0) | 0 (0) |  |
| Current (not daily) | 0 (0) | 0 (0) | 0 (0) | 0 (0) |  |  | 0 (0) | 0 (0) | 0 (0) | 0 (0) |  |
| Current (daily) | 6 (11.8) | 2 (28.6) | 0 (0) | 0 (0) |  |  | 3 (8.8) | 2 (66.7) | 0 (0) | 0 (0) |  |
| Drug use, *n* (%) ^e^ |  |  |  |  |  |  |  |  |  |  |  |
| Never | 35 (68.6) | 5 (71.4) | 2 (40.0) | 8 (80.0) | 0.169 |  | 21 (61.8) | 2 (66.7) | 2 (50.0) | 3 (100.0) | 0.483 |
| Former | 12 (23.5) | 0 (0) | 1 (20.0) | 2 (20.0) |  |  | 10 (29.4) | 0 (0) | 1 (25.0) | 0 (0) |  |
| Current (not daily) | 3 (5,9) | 2 (28.6) | 2 (40.0) | 0 (0) |  |  | 2 (5.9) | 1 (33.3) | 1 (25.0) | 0 (0) |  |
| Current (daily) | 1 (2.0) | 0 (0) | 0 (0) | 0 (0) |  |  | 1 (2.9) | 0 (0) | 0 (0) | 0 (0) |  |
| Cannabis use, *n* (%) |  |  |  |  |  |  |  |  |  |  |  |
| Never | 40 (81.6) | 5 (71.4) | 3 (60.00 | 8 (88,9) | 0.170 |  | 25 (75.8) | 2 (66.7) | 2 (50.0) | 3 (100.0) | 0.363 |
| Former | 7 (14.3) | 0 (0) | 1 (20.0) | 1 (11.1) |  |  | 6 (18.2) | 0 (0) | 1 (25.0) | 0 (0) |  |
| Current (not daily) | 1 (2,0) | 2 (28.6) | 1 (20.0) | 0 (0) |  |  | 1 (3.0) | 1 (33.3) | 1 (25.0) | 0 (0) |  |
| Current (daily) | 1 (2.0) | 0 (0) | 0 (0) | 0 (0) |  |  | 1 (3.0) | 0 (0) | 0 (0) | 0 (0) |  |
| Diet score, mean (SD) ^f^ | 6.3 (1.8) | 5.9 (2.2) | 5.8 (1.9) | 7.0 (2.4) | 0.512 |  | 6.4 (1.8) | 7.3 (0.6) | 6.5 (1.7) | 9.0 (1.7) | 0.222 |
| Active physically, *n* (%) ^g^ | 33 (64.7) | 4 (57.1) | 2 (40.0) | 7 (70.0) | 0.682 |  | 24 (70.6) | 2 (66.7) | 2 (50.0) | 2 (66.7) | 0.910 |
| *Medical parameters* |  |  |  |  |  |  |  |  |  |  |  |
| Disease extent at diagnosis, *n* (%) ^e^ |  |  |  |  |  |  |  |  |  |  |  |
| E1−Proctitis | 3 (7.9) | 3 (60.0) | 0 (0) | 2 (20.0) | 0.090 |  | 1 (3.7) | 1 (50.0) | 0 (0) | 0 (0) | 0.428 |
| E2−Left-sided colitis | 18 (47.4) | 2 (40.0) | 1 (50.0) | 4 (40.0) |  |  | 14 (51.9) | 1 (50.0) | 1 (33.3) | 2 (66.7) |  |
| E3−Pancolitis | 17 (44.7) | 0 (0) | 1 (50.0) | 4 (40.0) |  |  | 12 (44.4) | 0 (0) | 2 (66.7) | 1 (33.3) |  |
| Comorbidities, *n* (%) |  |  |  |  |  |  |  |  |  |  |  |
| Gastrointestinal | 36 (70.6) | 7 (100.0) | 3 (60.0) | 8 (80.0) | 0.325 |  | 23 (67.6) | 3 (100.0) | 2 (50.0) | 2 (66.7) | 0.734 |
| Musculoskeletal | 32 (62.7) | 4 (57.1) | 3 (60.0) | 7 (70.0) | 0.944 |  | 19 (55.9) | 2 (66.7) | 3 (75.0) | 2 (66.7) | 0.927 |
| Allergies | 28 (54.9) | 4 (57.1) | 3 (60.0) | 5 (50.0) | 1.000 |  | 17 (50.0) | 2 (66.7) | 4 (100.0) | 2 (66.7) | 0.304 |
| Cardiovascular | 27 (52.9) | 5 (71.4) | 3 (60.0) | 4 (40.0) | 0.647 |  | 19 (55.9) | 2 (66.7) | 3 (75.0) | 2 (66.7) | 0.927 |
| Mental Health | 26 (51.0) | 3 (42.9) | 3 (60.0) | 5 (50.0) | 1.000 |  | 17 (50.0) | 2 (66.7) | 2 (50.0) | 2 (66.7) | 1.000 |
| Respiratory | 25 (49.0) | 2 (28.6) | 4 (80.0) | 4 (40.0) | 0.372 |  | 18 (52.9) | 1 (33.3) | 2 (50.0) | 1 (33.3) | 0.880 |
| Urogenital | 26 (51.0) | 3 (42.9) | 3 (60.0) | 5 (50.0) | 1.000 |  | 15 (44.1) | 2 (66.7) | 3 (75.0) | 2 (66.7) | 0.536 |
| Metabolic | 18 (35.3) | 2 (28.6) | 1 (20.0) | 4 (40.0) | 0.915 |  | 10 (29.4) | 2 (66.7) | 2 (50.0) | 1 (33.3) | 0.538 |
| Hepatic | 15 (29.4) | 4 (57.1) | 2 (40.0) | 3 (30.0) | 0.532 |  | 9 (26.5) | 1 (33.3) | 1 (25.0) | 1 (33.3) | 1.000 |
| Cancer | 8 (15.7) | 1 (14.3) | 1 (20.0) | 0 (0) | 0.531 |  | 2 (5.9) | 1 (33.3) | 0 (0) | 0 (0) | 0.379 |
| Nervous system | 5 (9.8) | 2 (28.6) | 0 (0) | 1 (10.0) | 0.340 |  | 3 (8.8) | 1 (33.3) | 0 (0) | 0 (0) | 0.482 |
| Other | 31 (60.8) | 5 (71.4) | 4 (80.0) | 4 (40.0) | 0.449 |  | 19 (55.9) | 2 (66.7) | 3 (75.0) | 1 (33.3) | 0.766 |
| *Treatment history* |  |  |  |  |  |  |  |  |  |  |  |
| Length of exposure, mean (months, SD) | 100.5 (53.1) | 8.0 (4.5)* | 67.4 (31.1) | 32.4 (29.8)* | **<0.0001** |  | 113.4 (57.3) | 5.7 (4.0)* | 49.0 (28.9) | 32.3 (22.4) | **0.001** |
| Naïve to biologics, *n* (%) ^h^ | 47 (92.2) | 6 (85.7) | 5 (100.0) | 10 (100.0) | 0.677 |  | 29 (85.3) | 1 (33.3) | 4 (100.0) | 1 (33.3) | **0.032 ^k^** |
| First biologics taken, *n* (%) ^h^ |  |  |  |  |  |  |  |  |  |  |  |
| Adalimumab | 15 (29.4) | 4 (57.1) | 2 (40.0) | 8 (80.0) | 0.055 |  | 1 (2.9) | 1 (33.3) | 0 (0) | 2 (66.7) | **0.020 ^i^** |
| Infliximab | 28 (54.9) | 1 (14.3) | 3 (60.0) | 1 (10.0) |  |  | 29 (85.3) | 1 (33.3) | 4 (100.0) | 1 (33.3) |  |
| Vedolizumab | 4 (7.8) | 1 (14.3) | 0 (0) | 0 (0) |  |  | 4 (11.8) | 1 (33.3) | 0 (0) | 0 (0) |  |
| Golimumab | 4 (7.8) | 1 (14.3) | 0 (0) | 1 (10.0) |  |  |  |  |  |  |  |
| Number of biologics, *n* (%) ^h^ |  |  |  |  |  |  |  |  |  |  |  |
| Before drug initiation |  |  |  |  |  |  |  |  |  |  |  |
| 0 | 47 (92.2) | 6 (85.7) | 5 (100.0) | 10 (100.0) | 0.711 |  | 29 (85.3) | 1 (33.3) | 4 (100.0) | 1 (33.3) | **0.048 ^i^** |
| 1 | 3 (5.9) | 1 (14.3) | 0 (0) | 0 (0) |  |  | 3 (8.8) | 2 (66.7) | 0 (0) | 1 (33.3) |  |
| 2 | 1 (2.0) | 0 (0) | 0 (0) | 0 (0) |  |  | 2 (5.9) | 0 (0) | 0 (0) | 1 (33.3) |  |
| Total during UC treatment |  |  |  |  |  |  |  |  |  |  |  |
| 1 | 44 (86.3) | 0 (0) *^3,4^ | 0 (0) *^5,6^ | 2 (20.0) *^7^ | **<0.0001** |  | 27 (79.4) | 0 (0) | 0 (0) | 0 (0) | **<0.0001 ^i^** |
| 2 | 5 (9.8) | 4 (57.1) *^3^ | 3 (60.0) *^5^ | 3 (30.0) |  |  | 3 (8.8) | 1 (33.3) | 2 (50.0) | 0 (0) |  |
| 3 or more | 2 (3.9) | 3 (42.9) *^4^ | 2 (40.0) *^6^ | 5 (50.0) *^7^ |  |  | 4 (11.8) | 2 (66.7) | 2 (50.0) | 3 (100.0) |  |
| Concomitant medication, *n* (%) |  |  |  |  |  |  |  |  |  |  |  |
| 5-ASA, n (%) |  |  |  |  |  |  |  |  |  |  |  |
| At initiation only | 11 (21.6) | 0 (0) | 1 (20.0) | 3 (30.0) | 0.525 |  | 7 (20.6) | 0 (0) | 0 (0) | 0 (0) | 0.256 |
| During follow-up only | 7 (13.7) | 1 (14.3) | 1 (20.0) | 0 (0) |  |  | 5 (14.7) | 0 (0) | 2 (50.0) | 1 (33.3) |  |
| At initiation and during follow-up | 7 (13.7) | 2 (28.6) | 2 (40.0) | 2 (20.0) |  |  | 7 (20.6) | 2 (66.7) | 2 (50.0) | 1 (33.3) |  |
| No use | 26 (51.0) | 4 (57.1) | 1 (20.0) | 5 (50.0) |  |  | 15 (44.1) | 1 (33.3) | 0 (0) | 1 (33.3) |  |
| Corticosteroids, n (%) |  |  |  |  |  |  |  |  |  |  |  |
| At initiation only | 14 (27.5) | 1 (14.3) | 1 (20.0) | 1 (10.0) | **0.002 ^i^** |  | 7 (20.6) | 0 (0) | 0 (0) | 0 (0) | 0.178 |
| During follow-up only | 6 (11.8) | 2 (28.6) | 3 (60.0) | 4 (40.0) |  |  | 5 (14.7) | 0 (0) | 2 (50.0) | 1 (33.3) |  |
| At initiation and during follow-up | 6 (11.8) | 4 (57.1) | 1 (20.0) | 2 (20.0) |  |  | 9 (26.5) | 3 (100.0) | 2 (50.0) | 1 (33.3) |  |
| No use | 25 (49.0) | 0 (0) | 0 (0) | 3 (30.0) |  |  | 13 (38.2) | 0 (0) | 0 (0) | 1 (33.3) |  |
| Immunosuppressant, n (%) |  |  |  |  |  |  |  |  |  |  |  |
| At initiation only | 13 (25.5) | 0 (0) | 0 (0) | 2 (20.0) | 0.430 |  | 11 (32.4) | 0 (0) | 1 (25.0) | 1 (33.3) | 0.702 |
| During follow-up only | 7 (13.7) | 0 (0) | 1 (20.0) | 1 (10.0) |  |  | 6 (17.6) | 0 (0) | 2 (50.0) | 0 (0) |  |
| At initiation and during follow-up | 2 (3.9) | 0 (0) | 1 (20.0) | 0 (0) |  |  | 2 (5.9) | 0 (0) | 0 (0) | 0 (0) |  |
| No use | 29 (56.9) | 7 (100.0) | 3 (60.0) | 7 (70.0) |  |  | 15 (44.1) | 3 (100.0) | 1 (25.0) | 2 (66.7) |  |
| Colectomy, *n* (%) | 2 (3.9) | 1 (14.3) | 1 (20.0) | 3 (30.0) * | **0.031** |  | 1 (2.9) | 1 (33.3) | 1 (25.0) | 1 (33.3) | **0.032 ^i^** |
| *Laboratory results* |  |  |  |  |  |  |  |  |  |  |  |
| CRP >5mg/L at diagnosis, *n* (%) ^c^ | 8 (72.7) | 1 (33.3) | 0 (0) | 4 (100.0) | 0.102 |  | 4 (66.7) | 1 (100.0) | 1 (50.0) | 1 (100.0) | 1.000 |
| CRP >5mg/L at drug initiation, *n* (%) ^c,j^ | 26 (72.2) | 4 (80.0) | 4 (80.0) | 5 (62.5) | 0.951 |  | 19 (82.6) | 3 (100.0) | 3 (75.0) | 2 (66.7) | 0.878 |
| Positive to anti-drug antibodies, *n* (%) ^c,k^ | 14 (53.8) | 0 (0) | 1 (33.3) | 1 (33.3) | 0.759 |  | 15 (53.6) | 0 (NA) | 1 (33.3) | 0 (0) | 0.498 |
| *Toxicity* |  |  |  |  |  |  |  |  |  |  |  |
| Adverse events, *n* (%) ^l^ | 39 (76.5) | 4 (57.1) | 3 (60.0) | 6 (60.0) | 0.453 |  | 28 (82.4) | 2 (66.7) | 3 (75.0) | 2 (66.7) | 0.488 |

Abbreviations used: CRP = C-reactive protein, IR = intermediate responders; NR = non-responders; R = responders; PNR = primary non-responders; SNR = secondary non-responders, SD = Standard deviation, TNF = Tumor necrosis factor.

^a^ Includes primary non-responders, secondary non-responders and intermediate responders.

^b^ P-value of Fisher exact test comparing responders and stratified phenotype of response (primary non-responders, secondary non-responders and intermediate responders). *Post hoc* tests compare responders and each stratified phenotype of nonresponse separately for each pair of categories of the studied variable. Bold numbers indicate significance of Fisher test and asterisks (*) indicate significance of Fisher *post-hoc*. Pair of categories are identified by same number beside the asterisk.

^c^ Proportion/mean/SD calculated on available data.

^d^ Moderate use is considered as less than 15 drinks per week and excessive use is considered as higher than 15 drinks per week, as per Quebec Government’s recommendations.

^e^ Drugs included: cannabis, cocaine, methamphetamine, amphetamine, phencyclidine, codeine, lysergic acid diethylamide, psilocybin and psilocin.

^f^ Self-assessed score based on attention paid to alimentation at study inclusion (1 = no attention, 10 = greatest attention).

^g^ Active is defined as >150 min of moderate activity or >75 min of intense activity per week (World Health Organisation criteria).

^h^ Includes biologics and tofacitinib, a small molecule used as second line medication in ulcerative colitis.

^i^ No significant categories following fisher *post hoc* tests.

^j^ CRP within 4 months before molecule initiation.

^k^ Anti-drug antibodies level of 10 AU/mL or more.

^l^ At least one adverse event self-reported or collected in the medical chart.

**Table S4.** **Distribution of adverse events between stratified response’s phenotypes to anti-TNF and infliximab users in ulcerative colitis treatment**

|  | **All anti-TNF combined group** | | | | | |  | **Infliximab subgroup** | | | | | | |
| --- | --- | --- | --- | --- | --- | --- | --- | --- | --- | --- | --- | --- | --- | --- |
|  |  | **Phenotype stratification** | | |  | |  |  | **Phenotype stratification** | | | |  | |
|  | **R**  **(n = 51)** | **PNR**  **(n = 7)** | **SNR**  **(n = 5)** | **IR**  **(n = 10)** | **p-value ^b^** | |  | **R**  **(n = 34)** | **PNR**  **(*n* = 3)** | **SNR**  **(*n* = 4)** | **IR**  **(*n* = 3)** | | **p-value ^b^** | |
|  |  |  |  |  | **Un-adj** | **Adj** |  |  |  |  |  |  | **Un-adj** | **Adj** |
| Neurological ^c^ | 18 (35.3) | 1 (14.3) | 1 (20.0) | 2 (20.0) | 0.618 | 1.000 |  | 14 (41.2) | 0 (0) | 1 (25.0) | 1 (33.3) | 0.754 | | 1.000 |
| Skin ^d^ | 14 (27.5) | 1 (14.3) | 2 (40.0) | 4 (40.0) | 0.627 | 1.000 |  | 9 (26.5) | 0 (0) | 1 (25.0) | 1 (33.3) | 0.915 | | 1.000 |
| Musculoskeletal ^e^ | 8 (15.7) | 1 (14.3) | 1 (20.0) | 1 (10.0) | 1.000 | 1.000 |  | 6 (17.6) | 0 (0) | 1 (25.0) | 0 (0) | 1.000 | | 1.000 |
| Site injection ^f^ | 6 (11.8) | 1 (14.3) | 0 (0) | 4 (40.0) | 0.121 | 0.848 |  | 0 (0) | 0 (0) | 0 (0) | 0 (0) | 1.000 | | 1.000 |
| Infections ^g^ | 10 (19.6) | 0 (0) | 0 (0) | 0 (0) | 0.311 | 1.000 |  | 7 (20.6) | 0 (0) | 0 (0) | 0 (0) | 1.000 | | 1.000 |
| Gastrointestinal ^h^ | 5 (9.8) | 0 (0) | 0 (0) | 1 (10.0) | 1.000 | 1.000 |  | 4 (11.8) | 0 (0) | 0 (0) | 0 (0) | 1.000 | | 1.000 |
| Cardiovascular ^i^ | 3 (5.9) | 0 (0) | 1 (20.0) | 0 (0) | 0.445 | 1.000 |  | 2 (5.9) | 0 (0) | 1 (25.0) | 0 (0) | 0.548 | | 1.000 |
| Respiratory ^j^ | 3 (5.9) | 0 (0) | 0 (0) | 1 (10.0) | 0.770 | 1.000 |  | 3 (8.8) | 0 (0) | 0 (0) | 1 (33.3) | 0.482 | | 1.000 |
| Other ^k^ | 27 (52.9) | 3 (42.9) | 3 (60.0) | 5 (50.0) | 0.974 | 1.000 |  | 22 (64.7) | 2 (66.7) | 2 (50.0) | 2 (66.7) | 0.920 | | 1.000 |

Abbreviations used: Adj = adjusted; CRP = C-reactive protein, IR = intermediate responders; NR = non-responders; R = responders; PNR = primary non-responders; SNR = secondary non-responders, SD = Standard deviation, TNF = Tumor necrosis factor; Un-adj = un-adjusted.

^a^ Proportion calculated on total available data.

^b^ Adjusted p-value by Holm-Bonferroni correction.

^c^ Includes vertigo, dizziness, and headaches.

^d^ Includes urticarial, psoriasis, eczema, erythema, pruritus, and dryness.

^e^ Includes cellulitis and recurrent infections.

^f^ Includes arthralgia and myalgia.

^g^ Includes redness, swelling, itching, heat, and pain at site injection.

^h^ Includes nausea, vomiting, and dyspepsia.

^i^ Includes peripheral edema, increased blood pressure, and palpitations.

^j^ Includes exacerbated asthma and dyspnea.

^k^ Includes asthenia, alopecia, numbness, nosebleed, shivers, and weight loss.

**Table S5. Dominant and recessive models of genotypes distribution between responders/non-responders to anti-TNF and infliximab in ulcerative colitis treatment**

| **Genotypes ^a^** | **All anti-TNF combined group** | | | | |  | **Infliximab subgroup** | | | | |
| --- | --- | --- | --- | --- | --- | --- | --- | --- | --- | --- | --- |
|  | **R**  **(n = 49)** | **NR ^b^**  **(n = 22)** | **p-value ^c^** | | |  | **R**  **(n = 33)** | **NR** ^b^  **(n = 10)** | **p-value ^c^** | | |
|  |  |  | **Un-adj** | | **Adj** |  |  |  | **Un-adj** | | **Adj** |
| *TNFAIP3* |  |  |  |  | |  |  |  |  |  | |
| rs6927172 |  |  |  |  | |  |  |  |  |  | |
| CC | 33 (67.3) | 13 (59.1) | 0.594 | 1.000 | |  | 22 (66.7) | 4 (40.0) | 0.158 | 1.000 | |
| CG or GG | 16 (32.7) | 9 (40.9) |  |  | |  | 11 (33.3) | 6 (60.0) |  |  | |
| CC or CG | 49 (100.0) | 22 (100.0) | 1.000 | 1.000 | |  | 33 (100.0) | 10 (100.0) | 1.000 | 1.000 | |
| GG | 0 (0) | 0 (0) |  |  | |  | 0 (0) | 0 (0) |  |  | |
| *TNFRSF1A* |  |  |  |  | |  |  |  |  |  | |
| rs4149570 |  |  |  |  | |  |  |  |  |  | |
| AA | 7 (14.3) | 3 (13.6) | 1.000 | 1.000 | |  | 5 (15.2) | 1 (10.0) | 1.000 | 1.000 | |
| AC or CC | 42 (85.7) | 19 (86.4) |  |  | |  | 28 (84.8) | 9 (90.0) |  |  | |
| AA or AC | 34 (69.4) | 12 (54.5) | 0.285 | 1.000 | |  | 22 (66.7) | 6 (60.0) | 0.719 | 1.000 | |
| CC | 15 (30.6) | 10 (45.5) |  |  | |  | 11 (33.3) | 4 (40.0) |  |  | |
| rs767455 |  |  |  |  | |  |  |  |  |  | |
| TT | 20 (40.8) | 5 (22.7) | 0.183 | 1.000 | |  | 11 (33.3) | 1 (10.0) | 0.237 | 1.000 | |
| TC or CC | 29 (59.2) | 17 (77.3) |  |  | |  | 22 (66.7) | 9 (90.0) |  |  | |
| TT or TC | 44 (89.8) | 16 (72.7) | 0.084 | 0.588 | |  | 28 (84.8) | 8 (80.0) | 0.656 | 1.000 | |
| CC | 5 (10.2) | 6 (27.3) |  |  | |  | 5 (15.2) | 2 (20.0) |  |  | |
| *TNFRSF1B* |  |  |  |  | |  |  |  |  |  | |
| rs1061622 |  |  |  |  | |  |  |  |  |  | |
| TT | 28 (57.1) | 10 (45.5) | 0.443 | 1.000 | |  | 24 (72.7) | 2 (20.0) | **0.007** | **0.049** | |
| TG or GG | 21 (42.9) | 12 (54.5) |  |  | |  | 9 (27.3) | 8 (80.0) |  |  | |
| TT or TG | 47 (95.9) | 21 (95.5) | 1.000 | 1.000 | |  | 33 (100.0) | 9 (90.0) | 0.233 | 1.000 | |
| GG | 2 (4.1) | 1 (4.5) |  |  | |  | 0 (0) | 1 (10.0) |  |  | |
| rs1061624 |  |  |  |  | |  |  |  |  |  | |
| AA | 10 (20.4) | 7 (31.8) | 0.370 | 1.000 | |  | 7 (21.2) | 3 (30.0) | 0.674 | 1.000 | |
| AG or GG | 39 (79.6) | 15 (68.2) |  |  | |  | 26 (78.8) | 7 (70.0) |  |  | |
| AA or AG | 27 (55.1) | 16 (72.7) | 0.196 | 1.000 | |  | 17 (51.5) | 8 (80.0) | 0.153 | 1.000 | |
| GG | 22 (44.9) | 6 (27.3) |  |  | |  | 16 (48.5) | 2 (20.0) |  |  | |
| rs3397^d^ |  |  |  |  | |  |  |  |  |  | |
| CC | 4 (8.5) | 0 (0) | 0.299 | 1.000 | |  | 2 (6.5) | 0 (0) | 1.000 | 1.000 | |
| CT or TT | 43 (91.5) | 22 (100.0) |  |  | |  | 29 (93.5) | 10 (100.0) |  |  | |
| CC or CT | 16 (34.0) | 6 (27.3) | 0.782 | 1.000 | |  | 8 (25.8) | 2 (20.0) | 1.000 | 1.000 | |
| TT | 31 (66.0) | 16 (72.7) |  |  | |  | 23 (74.2) | 8 (80.0) |  |  | |
| rs976881 |  |  |  |  | |  |  |  |  |  | |
| TT | 7 (14.3) | 3 (13.6) | 1.000 | 1.000 | |  | 5 (15.2) | 0 (0) | 0.320 | 1.000 | |
| TC or CC | 42 (85.7) | 19 (86.4) |  |  | |  | 28 (84.8) | 10 (100.0) |  |  | |
| TT or TC | 25 (51.0) | 13 (59.1) | 0.611 | 1.000 | |  | 19 (57.6) | 8 (80.0) | 0.276 | 1.000 | |
| CC | 24 (49.0) | 9 (40.9) |  |  | |  | 14 (42.4) | 2 (20.0) |  |  | |

Abbreviations used: Adj = adjusted; NR = non-responders; R = responders; *TNFAIP3* = Tumor necrosis factor alpha induced protein 3; *TNFRSF1A* = Tumor necrosis factor receptor super-family, member 1A; *TNFRSF1B* = Tumor necrosis factor receptor super-family, member 1B; Un-adj = un-adjusted.

^a^ Models are based on alternative allele from NCBI. Dominant model (AA vs. AB/BB) is presented first followed by the recessive model (AA/AB vs. BB).

^b^ Includes primary non-responders, secondary non-responders and intermediate responders.

^c^ Bold numbers indicate significance of Fisher test. Adjusted p-value by Holm-Bonferroni correction.

^d^ This variant has missing genotypes. Two missing genotypes for the anti-TNF group (R, n = 47; NR, n = 22), and one missing genotype for the infliximab group (R, n = 31; NR, n = 10).

**Table S6. Stratified phenotype distribution of genotypes between anti-TNF and infliximab in ulcerative colitis treatment**

| **Genotypes ^a^** | **All anti-TNF combined group** | | | | | | |  | **Infliximab subgroup** | | | | | | |
| --- | --- | --- | --- | --- | --- | --- | --- | --- | --- | --- | --- | --- | --- | --- | --- |
|  | **R**  **(n = 49)** | **Phenotype stratification** | | |  | **p-value ^b^** | |  | **R**  **(n = 33)** | **Phenotype stratification** | | |  | **p-value ^b^** | |
|  |  | **PNR**  **(n = 7)** | **SNR**  **(n = 5)** | **IR**  **(n = 10)** |  | **Un-adj** | **Adj** |  |  | **PNR**  **(n = 3)** | **SNR**  **(n = 4)** | **IR**  **(n = 3)** |  | **Un-adj** | **Adj** |
| *TNFAIP3* |  |  |  |  |  |  |  |  |  |  |  |  |  |  |  |
| rs6927172 |  |  |  |  |  |  |  |  |  |  |  |  |  |  |  |
| CC | 33 (67.3) | 4 (57.1) | 3 (60.0) | 6 (60.0) |  | 0.889 | 1.000 |  | 22 (66.7) | 1 (33.3) | 2 (50.0) | 1 (33.3) |  | 0.478 | 1.000 |
| CG | 16 (32.7) | 3 (42.9) | 2 (40.0) | 4 (40.0) |  |  |  |  | 11 (33.3) | 2 (66.7) | 2 (50.0) | 2 (66.7) |  |  |  |
| GG | 0 (0) | 0 (0) | 0 (0) | 0 (0) |  |  |  |  | 0 (0) | 0 (0) | 0 (0) | 0 (0) |  |  |  |
| CG or GG | 16 (32.7) | 3 (42.9) | 2 (40.0) | 4 (40.0) |  | 0.889 | 1.000 |  | 11 (33.3) | 2 (66.7) | 2 (50.0) | 2 (66.7) |  | 0.478 | 1.000 |
| CC or CG | 49 (100.0) | 7 (100.0) | 5 (100.0) | 10 (100.0) |  | 1.000 | 1.000 |  | 33 (100.0) | 3 (100.0) | 4 (100.0) | 3 (100.0) |  | 1.000 | 1.000 |
| *TNFRSF1A* |  |  |  |  |  |  |  |  |  |  |  |  |  |  |  |
| rs4149570 |  |  |  |  |  |  |  |  |  |  |  |  |  |  |  |
| AA | 7 (14.3) | 0 (0) | 1 (20.0) | 2 (20.0) |  | 0.348 | 1.000 |  | 5 (15.2) | 0 (0) | 1 (25.0) | 0 (0) |  | 0.961 | 1.000 |
| AC | 27 (55.1) | 4 (57.1) | 3 (60.0) | 2 (20.0) |  |  |  |  | 17 (51.5) | 2 (66.7) | 2 (50.0) | 1 (33.3) |  |  |  |
| CC | 15 (30.6) | 3 (42.9) | 1 (20.0) | 6 (60.0) |  |  |  |  | 11 (33.3) | 1 (33.3) | 1 (25.0) | 2 (66.7) |  |  |  |
| AC or CC | 42 (85.7) | 7 (100.0) | 4 (80.0) | 8 (80.0) |  | 0.623 | 1.000 |  | 28 (84.8) | 3 (100.0) | 3 (75.0) | 3 (100.0) |  | 0.818 | 1.000 |
| AA or AC | 34 (69.4) | 4 (57.1) | 4 (80.0) | 4 (40.0) |  | 0.289 | 1.000 |  | 22 (66.7) | 2 (66.7) | 3 (75.0) | 1 (33.3) |  | 0.847 | 1.000 |
| rs767455 |  |  |  |  |  |  |  |  |  |  |  |  |  |  |  |
| TT | 20 (40.8) | 0 (0) | 2 (40.0) | 3 (30.0) |  | **0.015** | 0.105 |  | 11 (33.3) | 0 (0) | 1 (25.0) | 0 (0) |  | 0.295 | 1.000 |
| TC | 24 (49.0) | 6 (85.7) | 3 (60.0) | 2 (20.0) |  |  |  |  | 17 (51.5) | 3 (100.0) | 3 (75.0) | 1 (33.3) |  |  |  |
| CC | 5 (10.2) | 1 (14.3) | 0 (0) | 5 (50.0) |  |  |  |  | 5 (15.2) | 0 (0) | 0 (0) | 2 (66.7) |  |  |  |
| TC or CC | 29 (59.2) | 7 (100.0) | 3 (60.0) | 7 (70.0) |  | 0.186 | 1.000 |  | 22 (66.7) | 3 (100.0) | 3 (75.0) | 3 (100.0) |  | 0.710 | 1.000 |
| TT or TC | 44 (89.8) | 6 (85.7) | 5 (100.0) | 5 (50.0) |  | **0.022** | 0.154 |  | 28 (84.8) | 3 (100.0) | 4 (100.0) | 1 (33.3) |  | 0.145 | 1.000 |
| *TNFRSF1B* |  |  |  |  |  |  |  |  |  |  |  |  |  |  |  |
| rs1061622 |  |  |  |  |  |  |  |  |  |  |  |  |  |  |  |
| TT | 28 (57.1) | 4 (57.1) | 1 (20.0) | 5 (50.0) |  | 0.418 | 1.000 |  | 24 (72.7) | 0 (0) | 1 (25.0) | 1 (33.3) |  | **0.005** | **0.035^c^** |
| TG | 19 (38.8) | 2 (28.6) | 4 (80.0) | 5 (50.0) |  |  |  |  | 9 (27.3) | 2 (66.7) | 3 (75.0) | 2 (66.7) |  |  |  |
| GG | 2 (4.1) | 1 (14.3) | 0 (0) | 0 (0) |  |  |  |  | 0 (0) | 1 (33.3) | 0 (0) | 0 (0) |  |  |  |
| TG or GG | 21 (42.9) | 3 (42.9) | 4 (80.0) | 5 (50.0) |  | 0.504 | 1.000 |  | 9 (27.3) | 3 (100.0) | 3 (75.0) | 2 (66.7) |  | **0.006** | **0.042^c^** |
| TT or TG | 47 (95.9) | 6 (85.7) | 5 (100.0) | 10 (100.0) |  | 0.472 | 1.000 |  | 33 (100.0) | 2 (66.7) | 4 (100.0) | 3 (100.0) |  | 0.140 | 0.980 |
| rs1061624 |  |  |  |  |  |  |  |  |  |  |  |  |  |  |  |
| AA | 10 (20.4) | 2 (28.6) | 2 (40.0) | 3 (30.0) |  | 0.846 | 1.000 |  | 7 (21.2) | 1 (33.3) | 1 (25.0) | 1 (33.3) |  | 0.690 | 1.000 |
| AG | 17 (34.7) | 3 (42.9) | 2 (40.0) | 4 (40.0) |  |  |  |  | 10 (30.3) | 2 (66.7) | 2 (50.0) | 1 (33.3) |  |  |  |
| GG | 22 (44.9) | 2 (28.6) | 1 (20.0) | 3 (30.0) |  |  |  |  | 16 (48.5) | 0 (0) | 1 (25.0) | 1 (33.3) |  |  |  |
| AG or GG | 39 (79.6) | 5 (71.4) | 3 (60.0) | 7 (70.0) |  | 0.586 | 1.000 |  | 26 (78.8) | 2 (66.7) | 3 (75.0) | 2 (66.7) |  | 0.746 | 1.000 |
| AA or AG | 27 (55.1) | 5 (71.4) | 4 (80.0) | 7 (70.0) |  | 0.640 | 1.000 |  | 17 (51.5) | 3 (100.0) | 3 (75.0) | 2 (66.7) |  | 0.458 | 1.000 |
| rs3397^d^ |  |  |  |  |  |  |  |  |  |  |  |  |  |  |  |
| CC | 4 (8.5) | 0 (0) | 0 (0) | 0 (0) |  | 0.728 | 1.000 |  | 2 (6.5) | 0 (0) | 0 (0) | 0 (0) |  | 0.712 | 1.000 |
| CT | 12 (25.5) | 3 (42.9) | 0 (0) | 3 (30.0) |  |  |  |  | 6 (19.4) | 1 (33.3) | 0 (0) | 1 (33.3) |  |  |  |
| TT | 31 (66.0) | 4 (57.1) | 5 (100.0) | 7 (70.0) |  |  |  |  | 23 (74.2) | 2 (66.7) | 4 (100.0) | 2 (66.7) |  |  |  |
| CT or TT | 43 (91.5) | 7 (100.0) | 5 (100.0) | 10 (100.0) |  | 1.000 | 1.000 |  | 29 (93.5) | 3 (100.0) | 4 (100.0) | 3 (100.0) |  | 1.000 | 1.000 |
| CC or CT | 16 (34.0) | 3 (42.9) | 0 (0) | 3 (30.0) |  | 0.499 | 1.000 |  | 8 (25.8) | 1 (33.3) | 0 (0) | 1 (33.3) |  | 0.675 | 1.000 |
| rs976881 |  |  |  |  |  |  |  |  |  |  |  |  |  |  |  |
| TT | 7 (14.3) | 1 (14.3) | 1 (20.0) | 1 (10.0) |  | 0.865 | 1.000 |  | 5 (15.2) | 0 (0) | 0 (0) | 0 (0) |  | 0.239 | 1.000 |
| TC | 18 (36.7) | 2 (28.6) | 3 (60.0) | 5 (50.0) |  |  |  |  | 14 (42.4) | 1 (33.3) | 4 (100.0) | 3 (100.0) |  |  |  |
| CC | 24 (49.0) | 4 (57.1) | 1 (20.0) | 4 (40.0) |  |  |  |  | 14 (42.4) | 2 (66.7) | 0 (0) | 0 (0) |  |  |  |
| TC or CC | 42 (85.7) | 6 (85.7) | 4 (80.0) | 9 (90.0) |  | 0.932 | 1.000 |  | 28 (84.8) | 3 (100.0) | 4 (100.0) | 3 (100.0) |  | 1.000 | 1.000 |
| TT or TC | 25 (51.0) | 3 (42.9) | 4 (80.0) | 6 (60.0) |  | 0.605 | 1.000 |  | 19 (57.6) | 1 (33.3) | 4 (100.0) | 3 (100.0) |  | 0.158 | 1.000 |

Abbreviations used: Adj = adjusted; NR = non-responders; R = responders; *TNFAIP3* = Tumor necrosis factor alpha induced protein 3; *TNFRSF1A* = Tumor necrosis factor receptor super-family, member 1A; *TNFRSF1B* = Tumor necrosis factor receptor super-family, member 1B; Un-adj = un-adjusted.

^a^ Models are based on alternative allele from NCBI. Codominant model (AA vs AB vs BB) is presented first followed by the dominant model (AA vs AB/BB) and by the recessive model (AA/AB vs BB).

^b^ Bold numbers indicate significance of Fisher test. Adjusted p-value by Holm-Bonferroni correction.

^c^ No significant categories following fisher *post hoc* tests.

^d^ This variant has missing genotypes. Two missing genotypes for the anti-TNF group (R, n = 47; NR, n = 22), and one missing genotype for the infliximab group (R, n = 31; NR, n = 10).

**References cited in Supplementary Materials**

1. Jang, D.-I. *et al.* The Role of Tumor Necrosis Factor Alpha (TNF-α) in Autoimmune Disease and Current TNF-α Inhibitors in Therapeutics. *Int J Mol Sci* **22**, 2719 (2021).

2. López-Hernández, R. *et al.* Genetic polymorphisms of tumour necrosis factor alpha (TNF-α) promoter gene and response to TNF-α inhibitors in Spanish patients with inflammatory bowel disease. *International Journal of Immunogenetics* **41**, 63–68 (2014).

3. Netz, U. *et al.* Genetic polymorphisms predict response to anti-tumor necrosis factor treatment in Crohn’s disease. *World Journal of Gastroenterology* **23**, 4958–4967 (2017).

4. Song, G. G. *et al.* Association between TNF-α (-308 A/G, -238 A/G, -857 C/T) polymorphisms and responsiveness to TNF-α blockers in spondyloarthropathy, psoriasis and Crohn’s disease: a meta-analysis. *Pharmacogenomics* **16**, 1427–1437 (2015).

5. Tong, Q. *et al.* Association of TNF-α polymorphism with prediction of response to TNF blockers in spondyloarthritis and inflammatory bowel disease: a meta-analysis. *Pharmacogenomics* **14**, 1691–1700 (2013).

6. González, S. *et al.* TNF-alpha -308A promoter polymorphism is associated with enhanced TNF-alpha production and inflammatory activity in Crohn’s patients with fistulizing disease. *Am J Gastroenterol* **98**, 1101–1106 (2003).

7. Faustman, D. & Davis, M. TNF receptor 2 pathway: drug target for autoimmune diseases. *Nat Rev Drug Discov* **9**, 482–493 (2010).

8. Bank, S. *et al.* Associations between functional polymorphisms in the NFκB signaling pathway and response to anti-TNF treatment in Danish patients with inflammatory bowel disease. *Pharmacogenomics J* **14**, 526–534 (2014).

9. Matsukura, H., Ikeda, S., Yoshimura, N., Takazoe, M. & Muramatsu, M. Genetic polymorphisms of tumour necrosis factor receptor superfamily 1A and 1B affect responses to infliximab in Japanese patients with Crohn’s disease. *Aliment Pharmacol Ther* **27**, 765–770 (2008).

10. Pierik, M. *et al.* Tumour necrosis factor-α receptor 1 and 2 polymorphisms in inflammatory bowel disease and their association with response to infliximab. *Alimentary Pharmacology & Therapeutics* **20**, 303–310 (2004).

11. Wang, G.-B., Li, C.-R., Yang, J., Wen, P.-Q. & Jia, S.-L. A regulatory polymorphism in promoter region of TNFR1 gene is associated with Kawasaki disease in Chinese individuals. *Hum Immunol* **72**, 451–457 (2011).

12. Qasem, A., Ramesh, S. & Naser, S. A. Genetic polymorphisms in tumour necrosis factor receptors (TNFRSF1A/1B) illustrate differential treatment response to TNFα inhibitors in patients with Crohn’s disease. *BMJ Open Gastroenterol* **6**, e000246 (2019).

13. Mascheretti, S. *et al.* Pharmacogenetic investigation of the TNF/TNF-receptor system in patients with chronic active Crohn’s disease treated with infliximab. *Pharmacogenomics J* **2**, 127–136 (2002).

14. Steenholdt, C. *et al.* Genetic polymorphisms of tumour necrosis factor receptor superfamily 1b and fas ligand are associated with clinical efficacy and/or acute severe infusion reactions to infliximab in Crohn’s disease. *Alimentary Pharmacology & Therapeutics* **36**, 650–659 (2012).

15. Medrano, L. M. *et al.* Role of TNFRSF1B polymorphisms in the response of Crohn’s disease patients to infliximab. *Hum Immunol* **75**, 71–75 (2014).

16. Salvador-Martín, S. *et al.* Genetic predictors of long-term response and trough levels of infliximab in crohn’s disease. *Pharmacol Res* **149**, 104478 (2019).

17. Salvador-Martín, S. *et al.* Genetic Predictors of Long-term Response to Antitumor Necrosis Factor Agents in Pediatric Inflammatory Bowel Disease. *J Pediatr Gastroenterol Nutr* **71**, 508–515 (2020).

18. Dixit, V. M. *et al.* Tumor necrosis factor-alpha induction of novel gene products in human endothelial cells including a macrophage-specific chemotaxin. *J Biol Chem* **265**, 2973–2978 (1990).

19. Verstrepen, L. *et al.* Expression, biological activities and mechanisms of action of A20 (TNFAIP3). *Biochem Pharmacol* **80**, 2009–2020 (2010).

20. Elsby, L. M. *et al.* Functional evaluation of TNFAIP3 (A20) in rheumatoid arthritis. *Clin Exp Rheumatol* **28**, 708–714 (2010).

21. Priem, D., van Loo, G. & Bertrand, M. J. M. A20 and Cell Death-driven Inflammation. *Trends Immunol* **41**, 421–435 (2020).

22. Tong, Q. *et al.* Association of TNF-α polymorphism with prediction of response to TNF blockers in spondyloarthritis and inflammatory bowel disease: a meta-analysis. *Pharmacogenomics* **14**, 1691–1700 (2013).

23. Jezernik, G., Gorenjak, M. & Potočnik, U. MIF Variant rs755622 Is Associated with Severe Crohn’s Disease and Better Response to Anti-TNF Adalimumab Therapy. *Genes (Basel)* **14**, 452 (2023).
